# Thermo-Optic Nanomaterial Fiber Hydrogen Sensor

**DOI:** 10.3390/nano15060440

**Published:** 2025-03-13

**Authors:** Xuhui Zhang, Liang Guo, Xinran Wei, Qiang Liu, Yuzhang Liang, Junsheng Wang, Wei Peng

**Affiliations:** 1 Liaoning Key Laboratory of Marine Sensing and Intelligent Detection, Dalian Maritime University, Dalian 116026, China; xhzhang@dlmu.edu.cn (X.Z.); gmm9102@dlmu.edu.cn (L.G.); 2 Information Science and Technology College, Dalian Maritime University, Dalian 116026, China; 3 School of Physics, Dalian University of Technology, Dalian 116024, China; dutweixinran@163.com (X.W.); yzliang@dlut.edu.cn (Y.L.); 4 School of Computer and Electronic Information, Nanjing Normal University, Nanjing 210023, China; qiangliu@njnu.edu.cn

**Keywords:** hydrogen sensor, platinum-loaded tungsten trioxide, fiber grating, thermo-optical effect, hydrogen detection

## Abstract

In the current energy transition procedure, the application prospect of hydrogen as a clean energy material has attracted much attention. However, the widespread use of hydrogen is also accompanied by safety hazards, and how to detect hydrogen safely and efficiently has become a research focus. In this paper, we propose a fiber-optic hydrogen sensor based on the thermo-optic effect and nanomaterials, which combines the unique advantages of fiber-optic grating and platinum-loaded tungsten trioxide and is capable of detecting hydrogen concentration with high sensitivity. The principle of this sensor is to absorb hydrogen molecules by nanomaterials and trigger the exothermic effect, which leads to grating period change and refractive index change in the fiber, thus modulating the resonant wavelength of grating. By monitoring the wavelength drift in real time, the hydrogen concentration can be accurately detected. The experimental results show that the sensor can provide high sensitivity, fast response, wide detection range, and miniaturized design, which are suitable for hydrogen detection in complex environments. In addition, its dual-channel operational method further improves detection accuracy and environmental adaptability. This work provides technical support for safe hydrogen detection, which is suitable for hydrogen production, storage, industrial safety and environmental monitoring.

## 1. Introduction

As a clean and renewable energy material, hydrogen has received widespread attention in contemporary energy transition and environmental protection. Currently, hydrogen is considered an important choice for future energy because its combustion product is only water, which produces almost no environmental pollution. With the advancement of technology, hydrogen can not only be used in fuel cells but also as a reducing agent for transition metal catalysts, which have important applications in the chemical industry and environmental protection [1]. However, the widespread use of hydrogen is also accompanied by safety issues, and how to detect hydrogen safely and efficiently has become the focus of industry, transportation, and energy sectors.

Traditional hydrogen sensing technologies mainly include electrochemical sensors, etc. [2]. These technologies have certain limitations in terms of sensitivity, selectivity, stability, and long-term operation. Although electrochemical sensors have high sensitivity, their principle of operation involves the measurement of electrons, making them susceptible to electronic interference and toxicity, which limits their practical applications. Additionally, these conventional sensors have long response times, are susceptible to interference from other gases, and require periodic calibration and replacement of components, which increase the cost of use and maintenance effort [3]. Therefore, to overcome these limitations, researchers have begun to explore fiber-optic hydrogen sensing technologies based on nanomaterials [4,5,6]. Such technologies combine the high sensitivity of nanomaterials with the good transmission properties of fiber-optic materials, which not only improves the sensitivity of the sensor but also enhances stability.

In recent years, nanomaterials have been widely used in the hydrogen sensing field due to their unique physicochemical properties and developing a new chapter in hydrogen detection technology [7,8,9]. The introduction of nanomaterials has revolutionized the development of hydrogen sensing technology. The large surface area, excellent electronic conductivity, and tunable physicochemical properties of these materials have led to significant improvements in key performance indicators, such as sensitivity, selectivity, and detection limit, for hydrogen sensors based on nanomaterials. For example, by modifying specific functional groups on the surface of nanomaterials, the selectivity and sensitivity of hydrogen sensors can be significantly improved while maintaining long-term stability. Fiber-optic material, as an advanced sensing material with unique advantages—such as safety, less susceptibility to environmental influences, and remote sensing capability—provides a new way to detect hydrogen with high efficiency and accuracy [10,11,12]. When nanomaterials are combined with fiber-optic technology, not only do they retain their respective advantages but they also achieve complementary technologies, opening new paths for the development of hydrogen detection technology. Combining the unique properties of nanomaterials and the advanced nature of fiber-optic technology, a fiber-optic hydrogen sensor with high sensitivity, high selectivity, and high stability can be constructed.

In the design and synthesis of hydrogen-sensitive nanomaterials, researchers have made a lot of attempts and explorations. For example, the design and synthesis of hydrogen-sensitive materials using metal–organic frameworks, transition metal oxides, transition metal sulfides, etc. have made some progress [13,14,15]. These materials have not only improved the sensitivity of hydrogen detection but also demonstrated good performance in terms of stability and reproducibility. The characterization and performance evaluation of nanomaterials are key steps in the innovation and application of this technology. In order to gain an in-depth understanding of the properties of nanomaterials, their detailed characterization is required. This includes applying advanced characterization techniques like scanning electron microscopy to gather information on the morphology, chemical composition, and other properties of the nanomaterials [16,17,18]. These detailed characterization data are the basis for evaluating the performance of nanomaterials in hydrogen sensing applications. Conventional hydrogen sensing technologies, such as electrochemical sensors, although popular in applications, suffer from disadvantages such as susceptibility to electromagnetic interference, susceptibility to environmental conditions, and susceptibility to electrochemical substances. These problems limit the application range of conventional hydrogen sensors, especially the safety of their use in flammable and explosive environments. To overcome these limitations, research on fiber-optic hydrogen sensing technology has become an important scientific frontier. Fiber-optic sensors with their unique advantages—remote measurement, intrinsic safety, and high resistance to electromagnetic interference—offer new possibilities for hydrogen monitoring. Among them, fiber-optic hydrogen sensing technology using hydrogen-sensitive nanomaterials represents a major innovation in sensing technology. The development of hydrogen-sensitive nanomaterials provides new ways to improve the sensitivity and stability of hydrogen sensors. Through scientific design and synthesis of the structure and properties of nanomaterials, materials with specific hydrogen-sensitive properties can be obtained. These materials can cause changes in properties such as temperature or refractive index of the material when interacting with hydrogen, and these changes can be detected in real time and accurately by fiber-optic sensors [19,20,21,22]. The combination of nanomaterials and fiber-optic technology provides a new solution for hydrogen detection.

Therefore, this paper proposes a thermo-optic nanomaterial fiber-optic hydrogen sensor based on the hydrogen thermogenic effect, which combines the unique advantages of fiber-optic grating technology and nanomaterials and is capable of achieving highly sensitive detection of hydrogen concentration. The core principle is to absorb hydrogen molecules by nanomaterials and trigger the exothermic effect, which leads to a change in the fiber-optic material, thus changing the resonant wavelength of the grating. By monitoring the wavelength drift in real time, the hydrogen concentration can be accurately detected. The sensor offers several significant advantages, including high sensitivity and fast response, enabling rapid detection of trace amounts of hydrogen; a wide detection range, covering a wide range of applications from low to high concentrations; and a miniaturized and low-cost design, suitable for batch manufacturing and portable applications. In addition, the fiber grating is insensitive to electromagnetic interference, which, combined with the high temperature and corrosion resistance of the fiber itself, enables it to operate stably in complex environments. Its wide applicability covers hydrogen energy production and storage, industrial safety, environmental monitoring, and other fields, providing strong technical support for efficient and safe hydrogen detection.

## 2. Materials and Methods

**Figure 1:**
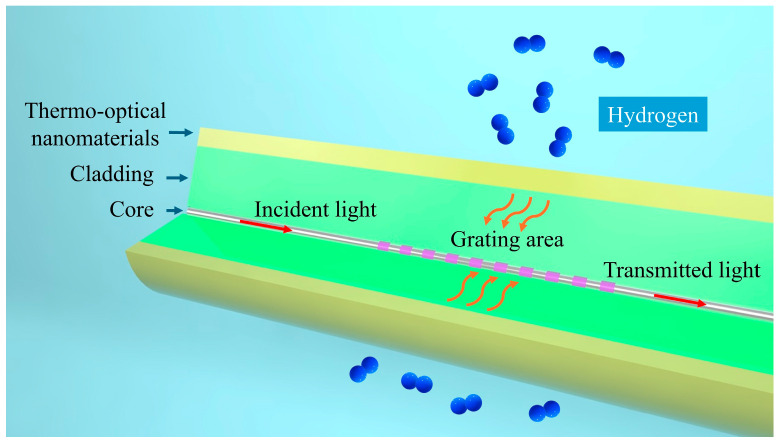
Schematic structure of thermo-optic nanomaterial fiber-optic hydrogen sensor.

Our proposed thermo-optic nanomaterial fiber-optic hydrogen sensor employs a long-period fiber grating as the fiber-optic material, which is used to realize optical sensing and fiber-optic communication of optical signals. As shown in Figure 1, the structure of the thermo-optic nanomaterial fiber-optic hydrogen sensor is shown. A long-period fiber grating is created by introducing a periodic variation in the refractive index along the core of a single-mode fiber. By adjusting the grating period, it is possible to match the phase between the core mode and the cladding mode for a specific wavelength. This phase matching causes light of that wavelength to couple into the cladding mode, resulting in resonance peaks in the transmission spectrum.

The following steps are required to fabricate a long-period fiber grating using the UV exposure method: first prepare a single-mode fiber, clean the fiber surface, and remove the coating layer to ensure that the UV light can act directly on the fiber core; then use a precision positioning device to fix the fiber and place a mask plate on it to form a periodic beam modulation. Typically, an excimer laser is chosen for the UV light source. After passing through the focusing system, the laser beam is adjusted to the proper spot size and collimated to irradiate the optical fiber. By precisely controlling the period of the mask and the exposure time of the laser, modulation of the refractive index is gradually created in the core of the fiber. Throughout the process, the laser power, the tensile state of the fiber, and the ambient temperature need to be tightly controlled to avoid additional effects on the fiber due to stress or thermal changes. After the exposure is completed, the optical fiber is placed in an appropriate position for a further refractive index modulation process. After completing the exposure, the fiber is removed from the fixture and its transmission spectrum is tested using a spectrometer to observe the central wavelength and intensity of the resonance peaks and to confirm that the grating period, depth, and performance meet the design requirements. If required, further optimization can be performed by re-exposure to adjust the resonance wavelength or improve the grating quality to meet the application requirements. The long-period fiber grating utilized in this study has a core diameter of approximately 9 μm, a cladding diameter of 125 μm, and a grating area length of 2 cm.

Further, we propose a fiber-optic hydrogen sensing technique based on platinum (Pt)-loaded tungsten trioxide (WO_3_) thermo-optical nanomaterials. The synthesis of WO_3_ nanomaterials can be achieved by several methods, including physical vapor deposition, the sol–gel method, and the hydrothermal method. Physical vapor deposition is a continuous process that can produce high-purity WO_3_ nanomaterials, but the equipment required for this method is costly, the operation is complicated, and the synthesis conditions are harsh. The sol–gel method can produce WO_3_ materials with uniform morphology and size and realize controlled production, however, its sol–gel conversion process is more complicated and the aging time is long, so it is difficult to promote its application on a large scale. In contrast, the hydrothermal method has the advantages of controllable morphology, simple equipment, convenient operation steps, and suitability for large-scale preparation.

In the application of Pt-WO_3_ in fiber-optic hydrogen sensors, the three-dimensional structure of WO_3_ nanoflakes is conducive to the deposition of Pt nanoparticles, and its porous structure can increase the contact area between the fiber-optic surface film and hydrogen and promote the adsorption and diffusion process of hydrogen on the surface of the WO_3_ material, which significantly improves the sensor’s hydrogen response performance. We obtained Pt-WO_3_ nanoflakes by a hydrothermal method, in which Pt was deposited on the surface of WO_3_ nanoflakes as an active component to catalyze the hydrogen reaction. The raw materials required for the experiment include sodium tungstate dihydrate, citric acid monohydrate, oxalic acid dihydrate, dilute hydrochloric acid, and platinum acetylacetonate.

The experimental steps mainly include: high-temperature reaction: transfer the configured solution into the polytetrafluoroethylene (PTFE) liner. Tighten the reactor and place it in a controllable oven for heating. The heating temperature is 160 °C. After the heating stops, wait for the kettle to cool down naturally. Centrifugation, washing, and drying: the obtained solution sample is transferred to a centrifuge tube for centrifugation. The precipitate obtained was washed with deionized water. The precipitate is transferred to a dish for drying to obtain WO_3_ powder. Mixing and grinding: a quantity of platinum acetylacetonate is weighed and mixed with tungsten trioxide made as described above. The mass ratio of platinum to tungsten is 1:5. Grind the powder several times using a grinding dish to mix it well. Sintering and preparation completion: transfer the mixed powder after grinding to a crucible. Place the crucible in a muffle furnace for sintering. After the heating is finished, the furnace is allowed to cool naturally to room temperature to obtain the Pt-deposited WO_3_ nanoflakes. Further, combine the Pt-loaded WO_3_ nanomaterials with long-period optical fiber grating to form a thermo-optic nanomaterial fiber-optic hydrogen sensor.

## 3. Results and Discussion

In this section, we discuss in detail the experimental results of fiber-optic hydrogen sensors based on thermo-optic effects and nanomaterials. First, we performed a detailed characterization of platinum-loaded tungsten trioxide (Pt-WO_3_) nanosheets and verified the structural and chemical compositions of the nanomaterials by scanning electron microscopy (SEM) and energy spectroscopy analysis. Subsequently, we demonstrated the spectral response characteristics of the sensor at different hydrogen concentrations and analyzed its performance in terms of sensitivity, response time, and recovery time. In addition, in order to further improve the anti-interference ability and environmental adaptability of the sensor, we proposed the design of a dual-channel long-period fiber grating hydrogen sensor and experimentally verified its stability and reliability under different environmental conditions. With these experimental results, we can comprehensively evaluate the performance of this sensor in hydrogen detection and provide strong technical support for its promotion in practical applications.

### 3.1. Characterization of Nanomaterials

**Figure 2:**
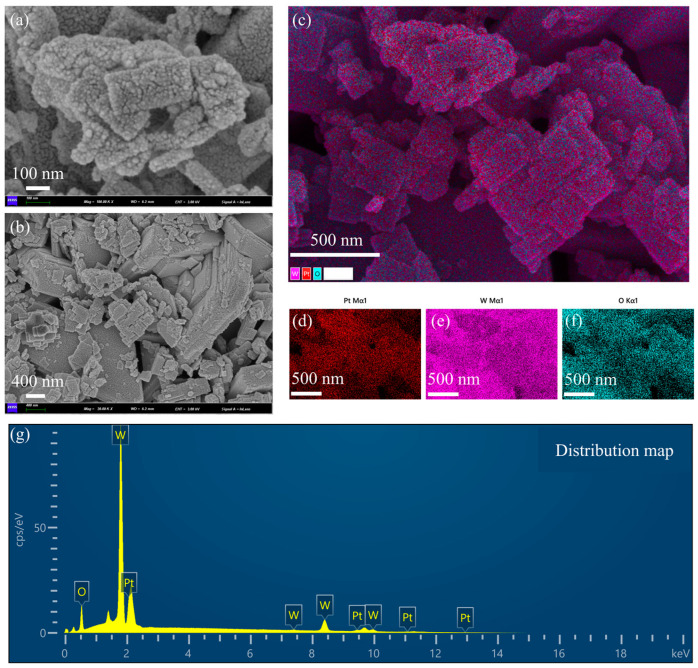
Scanning electron microscopy (SEM) and energy spectrum analysis of WO_3_ nanoflakes supported with Pt. (**a**) SEM image with higher magnification. (**b**) SEM image with low magnification. (**c**) The distribution of the stacking elements is shown. (**d**) Pt elemental distribution (red). (**e**) W elemental distribution (purple color). (**f**) Distribution of elemental O (blue-green color). (**g**) Energy spectra.

Scanning electron micrographs (SEMs) and energy spectrum analysis of Pt-loaded WO_3_ nanoflakes are shown in Figure 2. Figure 2a is an SEM image with higher magnification. The surface of the nanoflakes is rough with the presence of granular structures. These granular structures are Pt nanoparticles loaded on the surface of WO_3_. The edges of the nanoflakes are slightly curled, indicating that they have a large specific surface area, which is important for enhancing the catalytic performance. The rough surface structure helps to provide more active sites for Pt loading. The distribution of Pt particles was influenced by the surface energy of the WO_3_ sheet layer and the reduction conditions during the preparation process. Figure 2b is an SEM image with low magnification. The nanosheet material shows an irregular flake morphology with uneven size distribution of the flake layers. There is some stacking and overlapping between the nanoflakes, resulting in localized pore structures. The pores formed by the lamellae stacking will affect the gas transport properties of the material. The layer spacing and thickness of the lamellae can be tuned by the preparation process, which affects the Pt loading efficiency. Figure 2c is the distribution of the stacking elements. W (tungsten, purple), O (oxygen, blue-green), and Pt (platinum, red) are uniformly distributed, indicating that the loaded platinum particles are not significantly aggregated during the preparation process. The distribution areas of Pt particles overlap somewhat with those of tungsten and oxygen, suggesting that the Pt particles were deposited on the surface of the lamellae of tungsten trioxide. The uniformly distributed platinum benefits from the homogeneity of the precursor solution and the control of the reduction conditions. The interface between Pt particles and tungsten trioxide may provide abundant catalytic active sites suitable for chemocatalytic applications. Figure 2d–f are single-element distributions. Figure 2d shows the Pt elemental distribution (red): the Pt particles are distributed over a wide and uniform area, and no obvious aggregation of particles is observed, which suggests a more successful Pt loading process. The uniformity of Pt enhances the overall catalytic activity and reduces the formation of inactive regions. Figure 2e is W elemental distribution (purple color): tungsten shows a continuous distribution, indicating the structural integrity of the WO_3_ lamellae. The stability of the tungsten distribution region indicates that the WO_3_ matrix did not undergo significant structural changes during the Pt loading process. Figure 2f is the distribution of elemental O (blue-green color): the distribution of elemental oxygen is consistent with that of tungsten, indicating that the structure of WO_3_ is intact and its composition has not been changed by the Pt loading process. The abundance of oxygen may be important for the redox properties in the catalytic reaction. Figure 2g is the energy spectra. Oxygen (O): there is a clear peak in the low-energy region (~0.5 keV), indicating that elemental oxygen is one of the main components. Tungsten (W): the signal intensity of tungsten is the highest, which is shown as multiple peaks (~1.7 keV, ~8.4 keV, etc.), which is consistent with a high content of the WO_3_ matrix. Platinum (Pt): the characteristic peaks of platinum (~2 keV) are clearly visible, and the signal intensity is moderate, indicating moderate Pt loading. The presence of Pt indicates that the doping of the target substance was realized during the loading process, which has a key influence on the catalytic performance.

The lamellar structure of WO_3_ favors the increase in specific surface area and provides more Pt loading sites. The homogeneous distribution of the Pt particles indicates their potential as efficient catalysts. Pt-loaded WO_3_ materials are suitable for use in catalysis and gas sensors. The uniform distribution of Pt and the stability of the WO_3_ matrix may enhance the durability of the materials. Controlling the Pt particle size, a smaller particle size may further enhance the catalytic activity. Regulating nanosheet thickness and layer spacing, an optimized sheet structure may improve material transport properties. This set of images and data comprehensively demonstrates the microstructure and chemical composition of Pt-loaded WO_3_ nanoflakes. The uniform distribution of Pt, the lamellar structure of WO_3_, and the completeness of the elemental composition suggest that the material has excellent physicochemical properties and is suitable for catalytic and sensing applications.

### 3.2. Experimental Results of Hydrogen Detection

**Figure 3:**
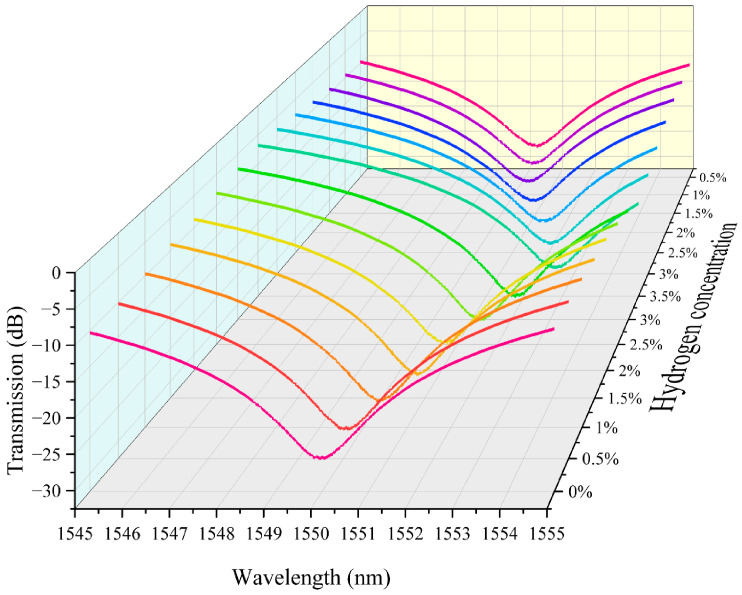
The characteristic spectra of the thermo-optic nanomaterial fiber-optic hydrogen sensor.

We integrated the above hydrogen exothermic nanomaterials with fiber grating into a sensor system and tested it to evaluate its performance. In this study we prepared housing for encapsulating the grating region of the sensor. It also serves the purpose of loading platinum-loaded tungsten trioxide. We controlled the loading amount in the grating region to 5 mg. The fiber-optic hydrogen sensing system based on thermo-optic nanomaterials is shown in Supplementary Materials Figure S15. In our experiments, we performed continuous repeatable detection of hydrogen gas in the near-explosive limit concentration range (0.5–3.5%). Air is the background gas used to control the hydrogen concentration in the gas mixture. The unit ppm is commonly used to express the concentration of a gas. It means parts per million. The conversion between ppm and percentage (%) is therefore 1% = 10,000 ppm. The characteristic spectra of the thermo-optic nanomaterial fiber-optic hydrogen sensor when the environment contains different concentrations of hydrogen are shown in Figure 3. As the hydrogen concentration increases, the transmission spectrum of the long-period fiber grating shifts towards the direction of wavelength increase. This indicates that the hydrogen exothermic effect leads to an increase in the temperature of the nanomaterial, which further increases the characteristic wavelength of the long-period fiber grating. This is because the characteristic wavelength of the long-period fiber grating is sensitive to temperature. The effect of hydrogen-induced heat production of nanomaterials leads to a change in the grating period and fiber refractive index, which modulates the resonance wavelength. Different hydrogen concentrations correspond to different wavelength shifts. When the hydrogen concentration is high, the wavelength shift is greater. This is since a high concentration of hydrogen generates more heat, which causes a more pronounced change in the grating period of the long-period fiber grating. It can be observed in the test data that the displacement of the spectral curve increases significantly at higher concentrations. The transmission spectra in the figure are regular, with the spectral curve gradually shifting to the right as the hydrogen concentration gradually increases from 0% to 3.5%, and the curves for each concentration are relatively independent, demonstrating good resolution. This indicates the response characteristics of the sensor, which can be used for quantitative measurement of hydrogen concentration.

**Figure 4:**
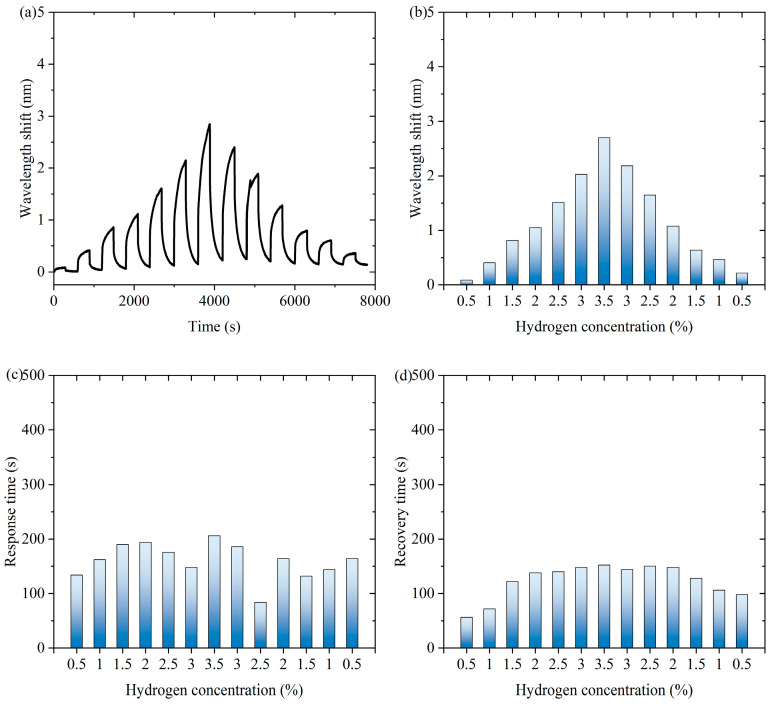
The experimental data of the fiber-optic hydrogen sensor for hydrogen thermophilic nanomaterials in the continuous switching between the states with and without hydrogen. (**a**) The characteristic wavelength shifts in the continuous experiment. (**b**) The characteristic wavelength shifts corresponding to the hydrogen concentrations. (**c**) The response time of the sensor to different hydrogen concentrations in continuous experiments. (**d**) The recovery time of the sensor from dehydrogenation in different hydrogen concentrations in continuous experiments.

Figure 4 shows experimental data of the fiber-optic hydrogen sensor for hydrogen thermophilic nanomaterials in the continuous switching between the states with and without hydrogen. Figure 4a shows the characteristic wavelength shift in the continuous experiment. The sensor exhibits a significant wavelength shift during the experiment. Over time, the wavelength shifts show periodic fluctuations under the influence of hydrogen concentration changes, which indicates that the sensor can sense the changes in hydrogen concentration stably. Figure 4b shows the characteristic wavelength shift corresponding to the hydrogen concentration during the process of first increasing and then decreasing hydrogen concentration. When the hydrogen concentration gradually increases and then decreases, the wavelength shift shows a trend consistent with the concentration change, which exhibits an approximately symmetric distribution, indicating that the wavelength response of the sensor is highly correlated with the hydrogen concentration. As can be seen from Supplementary Materials Figure S16, the response of the sensor during the change in hydrogen concentration is highly reversible, indicating that there is almost no hysteresis effect in the hydrogen absorption and desorption processes. When hydrogen is present in the air, the following chemical reactions occur in hydrogen-sensitive nanomaterials: WO_3_ + H_2_ → WO_2_ + H_2_O, WO_2_ + 1/2O_2_ → WO_3_. Hydrogen-induced exothermic nanomaterials release heat during the reaction process, which causes a temperature increase in the long-period fiber grating and changes its characteristic wavelength. After dehydrogenation, the reaction stops and the temperature decreases, so the proposed sensor has repeatability.

Figure 4c shows the response time of the sensor to different hydrogen concentrations in continuous experiments. The response time distribution of the sensor under different hydrogen concentrations is relatively uniform, showing that it can respond quickly when detecting different concentrations of hydrogen, and the shortest response time is 84 s, which indicates that its response performance is excellent and stable. Figure 4d shows the recovery time of the sensor from dehydrogenation at different hydrogen concentrations in successive experiments. The recovery time of the sensor from different hydrogen concentrations to a hydrogen-free state is relatively consistent, with the shortest recovery time of 56 s. This indicates that the sensor has good dehydrogenation recovery performance and can better adapt to changes in hydrogen concentration. Overall, the sensor shows good sensitivity and fast response and recovery performance in detecting changes in hydrogen concentration and can realize stable operation in a continuously switching environment, which is suitable for real-time hydrogen detection applications.

**Figure 5:**
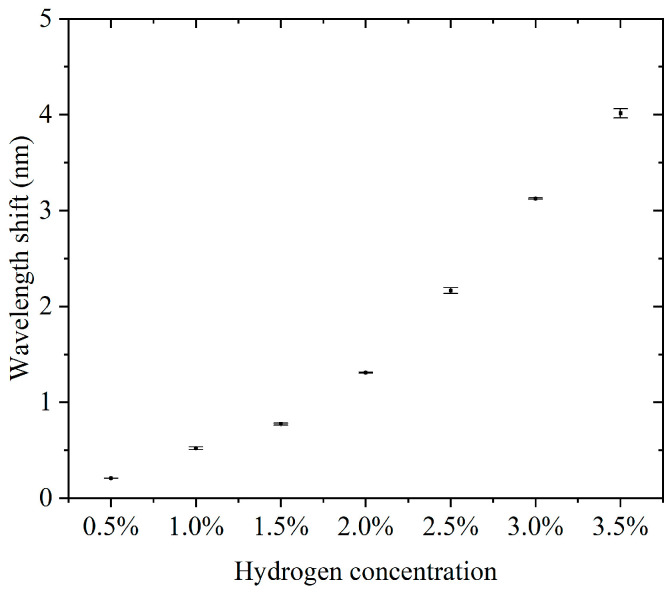
The relationship between hydrogen concentration and the characteristic wavelength offset.

Furthermore, we conducted continuous detection experiments using sensors in the 0.5–3.5% hydrogen concentration range. The concentration step interval was 0.5%. For each concentration, three consecutive cycles of hydrogen concentration detection and hydrogen desorption were performed. The results of the continuous detection experiments are presented in the Supplementary Materials. Supplementary Materials Figures S1–S7 show the results of successive hydrogen concentration detection cycles. Figure 5 shows the relationship between hydrogen concentration and the characteristic wavelength offset. The wavelength offset increases with increasing hydrogen concentration, and the response curve shows that the sensor has good sensitivity and stability in response to hydrogen concentration. The narrow range of the error bars indicates a high repeatability for each experiment. The errors did not show significant changes in each concentration, indicating that the sensor performance is highly consistent across different concentrations. The sensitivity, defined as the wavelength shift due to the change in concentration per unit of hydrogen, was about 3.807 nm for a total wavelength shift over the concentration range of 0.5% to 3.5%, corresponding to a 3% change in concentration and a sensitivity of 1.269 nm/%. This sensitivity indicates that the sensor has good detection performance over a wide concentration range. Compared to some fiber-optic hydrogen sensors, our proposed sensor has higher sensitivity in the operating interval [23,24,25]. At a low concentration (0.5%), the wavelength shift is about 0.208 nm, which indicates that the sensor has good responsiveness to low concentrations of hydrogen. The small error bar range indicates that the sensor has good stability and repeatability in the results in three tests. There was no significant difference in the error magnitude at each concentration, indicating that the sensor performance did not degrade with increasing hydrogen concentration. The sensor showed a consistent response at the same concentration, which is especially important for multiple tests. The sensor responded over a range of hydrogen concentrations from 0.5% to 3.5%, making it suitable for a wide range of concentrations. The wavelength shift of the sensor in this range is proportional to the hydrogen concentration. The sensitivity (1.269 nm/%) is sufficient to distinguish small changes in low-hydrogen-concentration environments and is suitable for leak detection or concentration monitoring. The small error range indicates that the sensor has good stability and reliability over many repeated uses. High sensitivity and response characteristics make it suitable for rapid detection of changes in hydrogen concentration. The ability to respond to lower concentrations of hydrogen is suitable for real-time monitoring of fuel cell operation.

**Figure 6:**
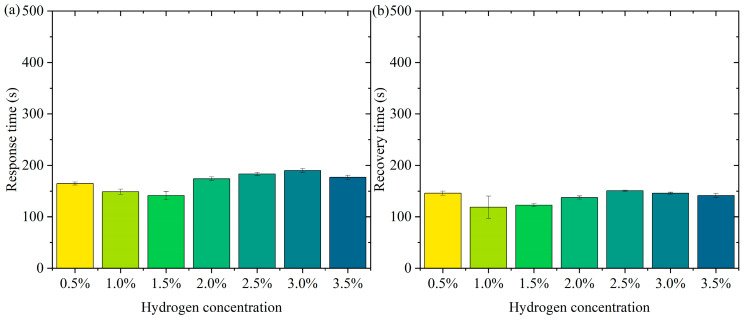
The pattern of change in response time and recovery time. (**a**) Response time. (**b**) Recovery time.

Figure 6 shows the pattern of change in response time and recovery time. Figure 6a is the pattern of change in response time. In the low-concentration range (0.5% to 1.5%), the response time decreases slightly (from about 165 s to 141 s). At higher concentrations (2.0% to 3.5%), the response time remains high but does not fluctuate much. The error bars show a small range of response times at all concentrations, indicating good repeatability and stability of the test results. In the lower concentration range (0.5% to 1.5%), the active sites of the sensor material (Pt-loaded WO_3_) adsorb hydrogen molecules rapidly at low hydrogen concentrations. The response time decreases slightly with increasing concentration, probably due to the higher transport rate and adsorption efficiency of hydrogen molecules. In the high-concentration range (2.0% to 3.5%), the occupancy of the sensor’s active sites gradually approaches saturation with increasing hydrogen concentration, limiting the adsorption/reaction process and leading to a slight increase in response time. The response time stabilized, indicating that the adsorption rate and sensing responsiveness of the material remained constant at higher concentrations. The error bars are short over all concentration ranges, indicating little dispersion in the test results. The test results are highly stable and reproducible. In practical applications, the sensor needs to show consistency over many repetitions of operation. The current data validates the reliability of the sensor. Over a wide concentration range (0.5% to 3.5%), the response time variation is small, indicating that the sensor has good dynamic response characteristics. The response time is controlled within a short period of time, which is suitable for real-time detection needs. The small error range indicates that the sensor performs consistently over multiple tests, making it suitable for high-frequency continuous monitoring. Fast response capability provides early warning at the beginning of a leak, suitable for hydrogen storage and transportation scenarios. Rapid detection capability for low-concentration hydrogen is suitable for real-time monitoring of fuel cell operation.

Figure 6b shows the pattern of change in recovery time. The recovery times were close over all concentration ranges (0.5% to 3.5%). The small margin of error indicates good reproducibility of the data. The recovery times were not significantly different in the low-concentration (0.5% to 1.5%) and high-concentration (2.0% to 3.5%) ranges. It indicates that the sensor shows stable desorption rate and material properties at different concentrations. On the surface of the sensor material, the detachment process of hydrogen molecules is mainly influenced by the material properties (e.g., specific surface area and active site distribution) and external conditions. In the tested concentration range, the variation of hydrogen concentration did not significantly affect the desorption kinetics, and therefore the recovery time behaved consistently. The Pt-loaded WO_3_ has good reversibility and the rate of desorption of hydrogen molecules from its surface may have reached a dynamic saturation state that does not change significantly with hydrogen concentration. The desorption process remains stable after the hydrogen concentration increases, although the occupancy rate of the adsorption sites may increase. The short error bars indicate that the dispersion between the results of the three tests at each concentration is small and the data are stable and reproducible. The sensor’s recovery performance over multiple tests was consistent and highly reliable. High repeatability is an important indicator of a sensor’s practical application, ensuring its consistent performance over long periods of use and multiple operations. The sensor therefore has recovery time stability, a characteristic that enables the sensor to recover quickly to its initial state in multiple consecutive tests, ready for the next test. The short error bars indicate that the dispersion of the experimental results is low, and the recovery performance of the sensor is stable across different test conditions. Recovery time stability makes it suitable for high-frequency detection requirements in hydrogen leakage monitoring systems. The recovery time consistency makes it suitable for real-time detection of hydrogen supply status in fuel cells.

Single-channel hydrogen sensors often suffer from poor anti-interference ability and poor environmental adaptability in complex environments, which limit their effectiveness in practical applications. To overcome these limitations, a dual-channel long-period fiber grating hydrogen sensor design based on thermo-optic nanomaterials is proposed in this paper. By introducing a dual-channel structure in the fiber grating sensor, dual monitoring of hydrogen concentration can be achieved, effectively improving the detection accuracy and environmental adaptability of the sensor. Specifically, the dual-channel design not only enhances the sensitivity of the sensor but also strengthens its fault-tolerance capability through redundant detection, ensuring reliability under different environmental conditions. This design scheme makes the sensor more suitable for complex practical application scenarios, especially in hydrogen detection systems that require high accuracy and reliability. The characteristic wavelength of the long-period fiber grating is closely related to the period length of the fiber grating. Therefore, we adjust the characteristic wavelength by adjusting the period length of the fiber grating, which in turn forms the second detection channel.

**Figure 7:**
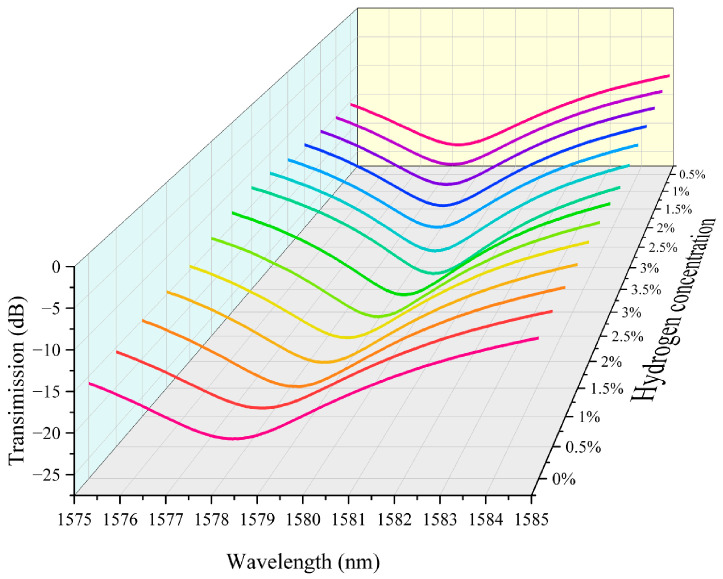
The experimental characteristic spectral variation of hydrogen detection in the second channel of a hydrogen sensor that integrates hydrogen exothermic nanomaterials with long-period fiber gratings.

Figure 7 shows the experimental characteristic spectral variation of hydrogen detection in the second channel of a hydrogen sensor that integrates hydrogen exothermic nanomaterials with long-period fiber gratings. As the hydrogen concentration increases, the transmission spectrum of the fiber grating undergoes a significant shift toward the long wavelength direction. This is due to the release of heat from the hydrogen exothermic nanomaterials when reacting with hydrogen, resulting in a change in the refractive index of the fiber grating, which leads to a shift of the spectral characteristic wavelength towards the long wavelength direction. When the hydrogen concentration is low (e.g., 0.5% or 1%), the wavelength shift is small, and the spectral shift is not obvious. In contrast, when the hydrogen concentration is high (e.g., 2% to 3%), the shift of the spectrum toward the long wavelength direction increases significantly, indicating that the change in hydrogen concentration is directly proportional to the amount of shift in the spectrum. The data in the figure indicate that the hydrogen sensor can effectively detect hydrogen concentration by monitoring the wavelength change of the spectrum, and the shift of the spectrum increases as the hydrogen concentration increases.

**Figure 8:**
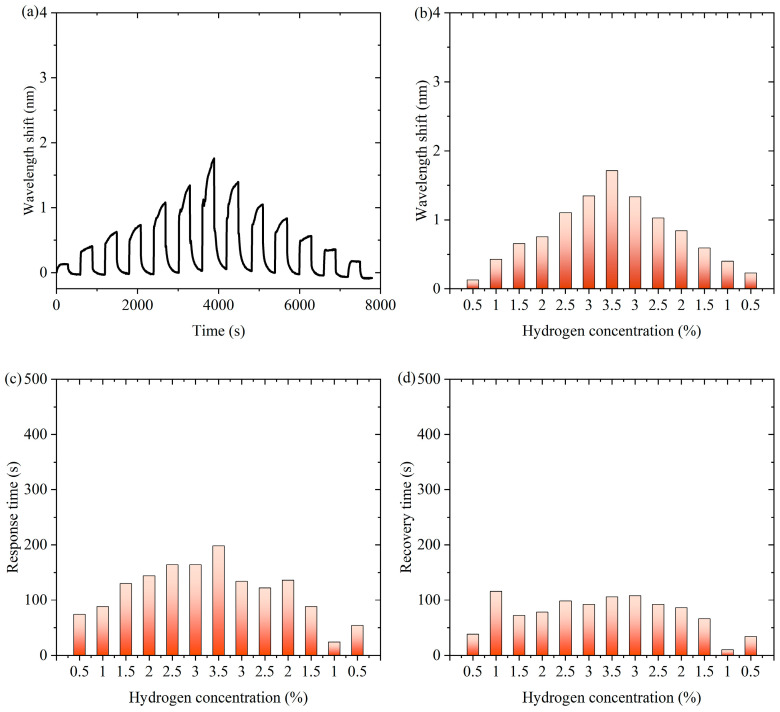
Experimental data for channel 2 of the thermo-optic nanomaterial fiber-optic hydrogen sensor in continuous switching between states with and without hydrogen. (**a**) The characteristic wavelength shift of the sensor in the continuous experiment. (**b**) The effect of hydrogen concentration changes on the wavelength shift of the sensor. (**c**) The response time of the sensor at different hydrogen concentrations. (**d**) The recovery time from different hydrogen concentrations.

Figure 8 is experimental data for channel 2 of the thermo-optic nanomaterial fiber-optic hydrogen sensor in continuous switching between states with and without hydrogen. Figure 8a shows the characteristic wavelength shift of the sensor in the continuous experiment, which appears to change periodically over time. This indicates that fluctuations in hydrogen concentration caused the sensor to respond in time. Figure 8b demonstrates the effect of hydrogen concentration changes on the wavelength shift of the sensor. As the hydrogen concentration gradually increases, the characteristic wavelength shift gradually increases and reaches a peak when the concentration reaches a maximum value, and then the wavelength shift gradually decreases as the hydrogen concentration decreases. A comparison of the responses for the gradually increasing and gradually decreasing phases of hydrogen concentration was obtained as shown in Supplementary Materials Figure S17. The curves for the rising and falling phases largely overlap, indicating that the response of the sensor is highly reversible. Figure 8c shows the response time of the sensor at different hydrogen concentrations. It can be observed that the response time of the sensor is shorter at lower hydrogen concentrations and longer at higher hydrogen concentrations. This may be related to the adsorption and reaction rate of hydrogen, and the diffusion and reaction process of hydrogen may take longer at higher concentrations, with the shortest response time being 24 s. Figure 8d, on the other hand, shows the recovery time from different hydrogen concentrations, with the shortest recovery time being 10 s.

**Figure 9:**
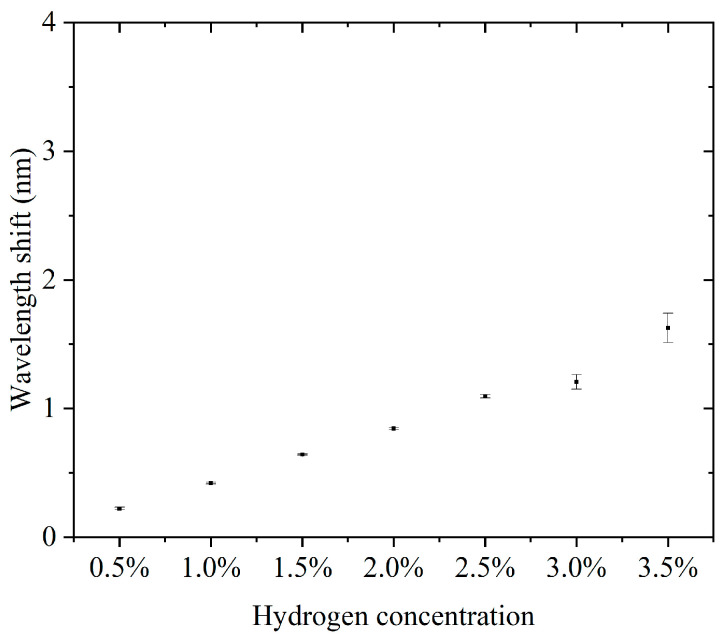
The hydrogen concentration versus the characteristic wavelength shift for the second detection channel of the fiber-optic hydrogen sensor with thermo-optic nanomaterials in three experiments.

Like the experimental results of channel 1, using channel 2 we performed a continuous cycle of hydrogen detection over a concentration range of 0.5–3.5% in steps of 0.5%, the results of which are presented in the Supplementary Materials. Supplementary Materials Figures S8–S14 show the results of the continuous hydrogen detection cycle. Figure 9 shows the hydrogen concentration versus the characteristic wavelength shift for the second detection channel of the fiber-optic hydrogen sensor with thermo-optic nanomaterials in three experiments. From the figure, there is an obvious positive correlation between hydrogen concentration and wavelength shift. The wavelength shift increased gradually with increasing hydrogen concentration, indicating a greater change in the optical response of the sensor at higher hydrogen concentrations. The error bars represent the measurement fluctuations for each data point in the experiment. From the figure, the error is relatively small, which means that the consistency of the experimental results is better, indicating that the reliability of the measurement process is higher. Since the error bars are small, it can be presumed that the measurement data are more accurate, indicating that the sensor is able to accurately detect changes in hydrogen concentration. Each increase in hydrogen concentration resulted in a large change in the wavelength offset. This indicates that the sensor has good sensitivity in the range of 0.5% to 3.5% hydrogen concentration.

**Figure 10:**
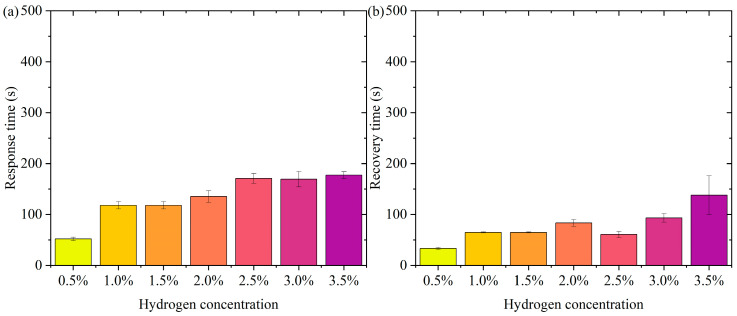
The hydrogen concentration versus response time/recovery time for the second detection channel of the hydrogen exothermic nanomaterial fiber-optic hydrogen sensor in three experiments. (**a**) Response time. (**b**) Recovery time.

Figure 10 shows the hydrogen concentration versus response time/recovery time for the second detection channel of the hydrogen exothermic nanomaterial fiber-optic hydrogen sensor in three experiments. The response time in Figure 10a shows an increasing trend with increasing hydrogen concentration. The response time at lower concentrations (e.g., 0.5%) is shorter, while the response time at higher concentrations (e.g., 3.5%) is relatively long. The relatively consistent size of the error bars at each concentration indicates that the experimental results are in good agreement, and the response time data at each concentration have similar standard deviations. It can be seen from the data that the response time of the sensor increased as the hydrogen concentration increased, which may be since higher concentrations of hydrogen take longer to react with the sensor’s reaction process. This trend suggests that, at higher concentrations of hydrogen, the sensor may take more time to complete the detection process or to process the changes in hydrogen, which may be related to factors such as the diffusion rate of hydrogen, the response characteristics of the sensor, and other factors.

Figure 10b shows the second detection channel of the hydrogen exothermic nanomaterial fiber-optic hydrogen sensor with hydrogen concentration versus recovery time in three experiments. From the 0.5% to 3.5% hydrogen concentration, the recovery time has an increasing trend. This trend indicates that the increase in hydrogen concentration influences the recovery process, probably since the process of desorption of hydrogen molecules from the surface of the sensor becomes slower when the hydrogen concentration increases. At low concentrations (e.g., 0.5% and 1%), the recovery time is shorter, and the error range is smaller. At high concentrations (e.g., 3.5%), the recovery time increases significantly, and the error range is relatively large, which may be because the desorption process becomes more complex and slower at higher concentrations due to higher hydrogen saturation on the sensor surface.

To investigate the temperature-sensitive characteristics of the sensor, we tested the response of the two channels of the sensor to changes in ambient temperature separately, as shown in Supplementary Materials Figure S18. The temperature test data further corroborate the working principle of the sensor, where the nanomaterials undergo hydrogen exotherm in hydrogen gas, causing the sensor spectrum to redshift.

## 4. Conclusions

This paper illustrated and demonstrated fiber-optic hydrogen sensing technology based on the thermo-optic effect and nanomaterials, which combines the advantages of fiber-optic grating technology and nanomaterials to achieve highly sensitive hydrogen concentration detection. The core principle of the sensor is that the nanomaterials absorb hydrogen molecules and trigger an exothermic effect, which leads to changes in the fiber-optic material, thus changing the resonance wavelength of the grating. By monitoring the wavelength drift in real time, the hydrogen concentration can be accurately detected. Experimental results show that the sensor has good sensitivity and stability in the range of 0.5% to 3.5% hydrogen concentration, and the wavelength drift is proportional to the hydrogen concentration. The response time and recovery time of the sensor showed good repeatability and stability at different concentrations.

In addition, the palladium (Pt)-loaded tungsten trioxide (WO_3_) nanosheets were also characterized in detail in the study, which showed that the Pt nanoparticles were uniformly distributed on the surface of the WO_3_ nanosheets, which contributed to the improvement of the catalytic performance. In order to improve the anti-interference ability and environmental adaptability of the sensor, a dual-channel long-period fiber-optic hydrogen sensor design based on thermo-optic nanomaterials is proposed in this paper, and the experimental results show that the design improves the sensitivity and fault tolerance of the sensor and ensures the reliability under different environmental conditions.

## Data Availability

The original contributions presented in this study are included in the article/Supplementary Materials. Further inquiries can be directed to the corresponding authors.

## Supplementary Materials

The following supporting information can be downloaded at: www.mdpi.com/article/10.3390/nano15060440/s1, Figure S1: The continuous repeatability detection of the sensor for 0.5% concentration of hydrogen gas; Figure S2: The continuous repeatability of the sensor for 1.0% concentration of hydrogen; Figure S3: The results of the continuous repeatability of the sensor for 1.5% concentration of hydrogen; Figure S4: The results of the continuous repeatability of the sensor for 2% concentration of hydrogen; Figure S5: The results of the continuous repeatability of the sensor for 2.5% hydrogen concentration; Figure S6: The results of the continuous repeatability of the sensor for 3% concentration of hydrogen; Figure S7: The results of the continuous repeatability of the sensor for a 3.5% concentration of hydrogen; Figure S8: The experimental data of the second channel of the fiber-optic hydrogen sensor with thermo-optic nanomaterials in 0.5% concentration of hydrogen; Figure S9: The experimental data for the second detection channel of the hydrogen exothermic nanomaterial fiber-optic hydrogen sensor in 1% concentration of hydrogen; Figure S10: The experimental data of the second detection channel of the hydrogen exothermic nanomaterial fiber-optic hydrogen sensor in 1.5% concentration of hydrogen; Figure S11: The experimental data for the second detection channel of the hydrogen exothermic nanomaterial fiber-optic hydrogen sensor in 2% concentration of hydrogen; Figure S12: The experimental data for the second detection channel of the hydrogen exothermic nanomaterial fiber-optic hydrogen sensor in 2.5% concentration of hydrogen; Figure S13: The experimental data for the second detection channel of the hydrogen exothermic nanomaterial fiber-optic hydrogen sensor in 3% concentration of hydrogen; Figure S14: The experimental data for the second detection channel of the hydrogen exothermic nanomaterial fiber-optic hydrogen sensor in 3.5% concentration hydrogen; Figure S15: Dual-channel fiber-optic hydrogen sensing test system based on thermo-optic nanomaterials; Figure S16: The characteristic wavelength shifts correspond to the hydrogen concentration; Figure S17: The effect of hydrogen concentration changes on the wavelength shifts of the sensor; Figure S18: Response of the two channels of the sensor to changes in ambient temperature.
