# Supplementary material for: Thermo-Optic Nanomaterial Fiber Hydrogen Sensor
Source: Nanomaterials (Basel). 2025 Mar 13;15(6):440. doi: 10.3390/nano15060440 (PMC11946813; doi:10.3390/nano15060440)
Supplement: Supplementary file 1 [file nanomaterials-15-00440-s001.zip › nanomaterials-3493503-supplementary.pdf]

nanomaterials

Supplementary Materials

Thermo-Optic Nanomaterial Fiber Hydrogen Sensor

Xuhui Zhang [1,2], Liang Guo [1,2], Xinran Wei [3], Qiang Liu [4], Yuzhang Liang [3], Junsheng Wang [1,2,*] and Wei Peng [3,*]

[1] Liaoning Key Laboratory of Marine Sensing and Intelligent Detection, Dalian Maritime University, Dalian 116026, China; xhzhang@dlmu.edu.cn (X.Z.); gmm9102@dlmu.edu.cn (L.G.)
[2] Information Science and Technology College, Dalian Maritime University, Dalian 116026, China
[3] School of Physics, Dalian University of Technology, Dalian 116024, China; dutweixinran@163.com (X.W.); yzliang@dlut.edu.cn (Y.L.)
[4] School of Computer and Electronic Information, Nanjing Normal University, Nanjing 210023, China; qiangliu@njnu.edu.cn
* Correspondence: wangjsh@dlmu.edu.cn (J.W.); wpeng@dlut.edu.cn (W.P.)

Academic Editor: Marco Cannas

Received: 8 February 2025
Revised: 11 March 2025
Accepted: 12 March 2025
Published: 13 March 2025

Citation: Zhang, X.; Guo, L.; Wei, X.; Liu, Q.; Liang, Y.; Wang, J.; Peng, W. Thermo-Optic Nanomaterial Fiber Hydrogen Sensor. *Nanomaterials* **2025**, *15*, 440. https://doi.org/10.3390/nano15060440

Copyright: © 2025 by the authors. Licensee MDPI, Basel, Switzerland. This article is an open access article distributed under the terms and conditions of the Creative Commons Attribution (CC BY) license (https://creativecommons.org/licenses/by/4.0/).

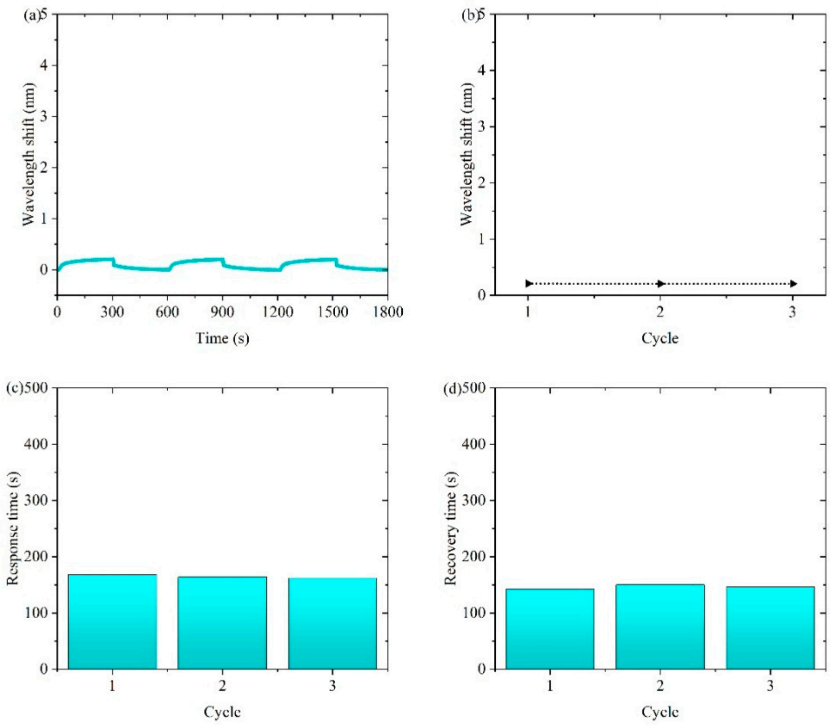

Figure S1. The continuous repeatability detection of the sensor for 0.5% concentration of hydrogen gas. (a) The results of the continuous test. (b) The wavelength response for three tests. (c) The response time for the three tests. (d) The recovery time for the three tests.

Figure S1 shows the results of the continuous repeatability detection of the sensor for a 0.5% concentration of hydrogen gas. Figure S1 (a) shows the results of the continuous test, which shows the wavelength shift over time for a test period of 1800 seconds. The magnitude of the wavelength shift remains consistent for each cycle, indicating a stable sensor response. The peak in the curve corresponds to the response process when the sensor detects hydrogen, followed by a gradual return to the baseline value, indicating a reversible response behavior of the sensor. This indicates high stability and good repeatability of the sensor under continuous use. Figure S1 (b) shows the wavelength

response for three tests. The wavelength offset values remain consistent over the three cyclic tests with an average offset of 0.208 nm, showing a high degree of repeatability. The wavelength offsets did not change significantly over the multiple tests, indicating that the sensor has good repeatability of the hydrogen detection results. This consistency indicates that the sensor is able to provide a reliable detection signal under the same test conditions. Repeatability is an important characteristic of the sensor, especially when used multiple times in industrial environments, and this performance ensures the long-term reliability of the sensor. Figure S1 (c) shows the response time for the three tests. The response time remained essentially the same over the three tests, with an average response time of 165 seconds. There is no noticeable drift or delay. The stability of the response time indicates that the sensor's speed of hydrogen detection has not been affected in multiple uses, which may be related to the good thermal conductivity and surface adsorption ability of the material. The response time is suitable for application scenarios such as hydrogen concentration detection environments in hydrogen-powered ships or hydrogen energy carriers. Figure S1 (d) shows the recovery time for the three tests. The recovery time is also consistent across the three tests with an average response time of 146 seconds, which is close to the response time. The recovery process was complete, indicating that the sensor was able to return to its initial state after each cycle. The stability of the recovery time indicates that the material is able to release hydrogen quickly after adsorption and that the active sites on the surface of the material are not damaged. The recovery time is close to the response time, indicating that the sensor can recover relatively quickly for the next detection.

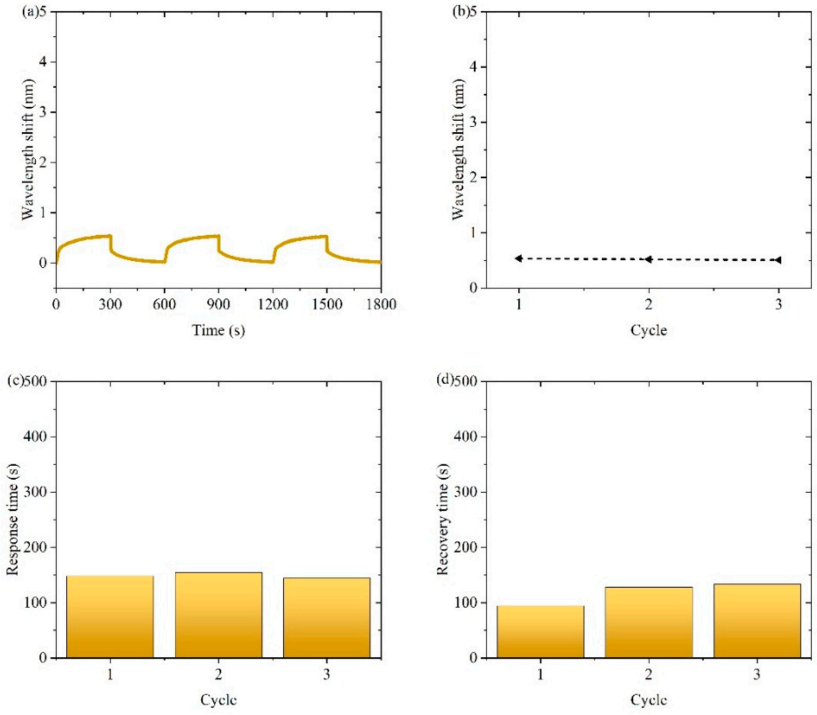

Figure S2. The continuous repeatability of the sensor for 1.0% concentration of hydrogen. (a) The results of the continuous test. (b) The wavelength response for three tests. (c) The response times for the three tests. (d) The recovery time for the three tests.

Figure S2 shows the results of the continuous repeatability of the sensor for 1.0% concentration of hydrogen. Figure S2 (a) shows the results of the continuous test. The

wavelength shift with time shows multiple response cycles, and good repeatability is presented in the curve, with the ability to recover to a position close to the initial baseline after each response. The maximum wavelength shift in the test is correlated with the hydrogen concentration (1%), and the shift amplitude is relatively stable, indicating that the material has a high sensitivity to the response to hydrogen concentration. The consistent behavior of the response in each cycle indicates that the sensor is more stable during continuous operation. The material has achieved a balance between high sensitivity and low drift, which is suitable for long-term online monitoring. Figure S2 (b) shows the wavelength response for three tests. The wavelength shift remains consistent in the three tests, with the maximum shift averaged at 0.520 nm. The data show good reproducibility, and the difference in wavelength shift between each test is negligible. The stability of the wavelength response indicates a high degree of consistency in the detection of the material at the same concentration of hydrogen (1%). This repeatability ensures the reliability of the sensor under the same test conditions for multiple reuse scenarios. Figure S2 (c) shows the response times for the three tests. Response times were consistent across the three tests, with an average response time of 149 seconds, which is slightly faster than the response time for low hydrogen concentrations (e.g., 0.5% concentration). No significant fluctuations or delays were observed in the data. The faster response time indicates that the material adsorbs and detects hydrogen more rapidly at a 1% hydrogen concentration. The stability of the response time further demonstrates the sensor's ability to respond instantaneously to changes in hydrogen concentration. Figure S2 (d) shows the recovery time for the three tests. The recovery time is also consistent across the three tests with an average recovery time of 119 seconds, which is comparable to the response time. The recovery behavior shows good stability and the difference between the data is negligible. The stability of the recovery time indicates that the material is well reversible during hydrogen desorption and is able to quickly recover from the detected state to the initial state. This fast recovery characteristic contributes to the rapid reuse of the sensor in continuous monitoring.

The wavelength shift amplitude remained stable at 1% hydrogen concentration, indicating good sensor sensitivity. No significant drift was observed in the test data, indicating that both sensitivity and stability were guaranteed. The response time (149 s) and recovery time (119 s) are shorter at a 1% hydrogen concentration, indicating that the increase in hydrogen concentration enhances the adsorption and desorption rate of the material. This fast response and recovery behavior is suitable for real-time monitoring of hydrogen leakage or concentration changes. The data in Figure 6 (b), (c), and (d) show that the wavelength response, response time, and recovery time of the sensor are highly reproducible over multiple tests, verifying the reversibility and long-term stability of the sensor.

Figure S3 shows the results of the continuous repeatability of the sensor for a 1.5% concentration of hydrogen. Figure S3 (a) shows the results of the continuous test, where the wavelength shift over time shows multiple cycles. The response of each cycle has good repeatability and the response peaks are followed by a recovery close to the initial baseline. The magnitude of the wavelength shift indicates that the sensor has good sensitivity at a 1.5% hydrogen concentration. The waveforms were nearly uniform from cycle to cycle, demonstrating the stability of the sensor in continuous monitoring. The material has high sensitivity and stable response, which makes it suitable for real-time monitoring in high-hydrogen-concentration environments. Figure S3 (b) shows the wavelength response of three tests. The wavelength shift values remained consistent with an average shift of 0.774 nm without significant fluctuations in the three cycles of testing. The wavelength response of the sensor is highly repeatable at a 1.5% hydrogen concentration. This good repeatability ensures that the sensor remains accurate over many

reuses and is suitable for long-term applications. Figure S3 (c) shows the response time for the three tests. The response time is consistent across the three tests with an average value of 141 seconds. The response time did not change significantly with increasing hydrogen concentration, indicating that the hydrogen adsorption process is less affected by the concentration change. The stability of the response time further validates the rapid detection capability of the sensor, which is suitable for real-time monitoring. Figure S3 (d) is recovery time for three tests. The average recovery time was 123 seconds and did not change significantly over the three tests. The stable recovery time indicates that the material is well reversible during hydrogen desorption and the hydrogen molecules can be quickly released from the material surface. The recovery process was complete and no residual response signal was observed, further demonstrating the reusability of the sensor.

This set of test data demonstrates that the hydrogen exothermic nanomaterial and fiber grating integrated sensor has excellent sensitivity, fast response capability, and good repeatability at a 1.5% hydrogen concentration. The sensor exhibits a consistent response signal with good stability and reversibility in continuous testing.

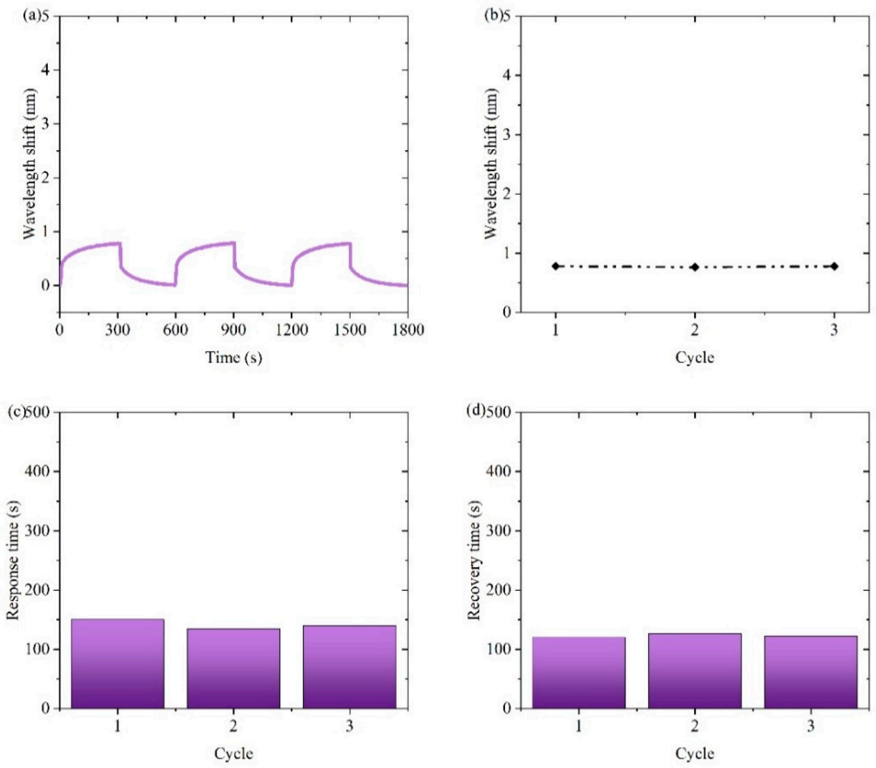

Figure S3. The results of the continuous repeatability of the sensor for 1.5% concentration of hydrogen. (a) The results of the continuous test. (b) The wavelength response of three tests. (c) The response time for the three tests. (d) The recovery time for three tests.

Figure S4 shows the results of the continuous repeatability of the sensor for a 2% concentration of hydrogen. Figure S4 (a) shows the results of continuous testing, where the wavelength shift over time exhibits three cycles, and in each cycle, the wavelength shift curve shows consistency and is able to return to the baseline level after the peak response. The magnitude of the wavelength shift remains stable at a 2% hydrogen concentration, indicating that the sensitivity of the sensor is not affected by a further increase in concentration. The shape of the curve is consistent with tests at low hydrogen

concentrations (e.g. 1% or 1.5%), indicating that the material maintains good performance at higher concentrations. The performance of the sensor was stable in continuous testing with no significant drift or degradation. Figure S4 (b) shows the wavelength response for the three tests. The wavelength shift maximum remained consistent over the three test cycles, averaging 1.310 nm. There were no significant fluctuations or anomalies, and the data repeatability was excellent. The repeatability of the wavelength shift indicates that the sensor's response to hydrogen concentration is stable and reliable. Figure S4 (c) shows the response times for the three tests. The response times were consistent over the three tests, averaging 174 seconds. The stability of the response time indicates that the increase in hydrogen concentration did not significantly affect the detection speed of the sensor. The adsorption process of hydrogen by the material is less affected by the concentration change. Figure S4 (d) shows the recovery times for the three tests. The recovery time was also consistent with an average of 137 seconds with no significant delay or residual signal during the recovery process. The stability of the recovery time indicates good reversibility of the material during hydrogen desorption. The sensor demonstrated the ability to recover quickly and return to baseline in a short period of time, ready for the next test cycle.

The wavelength shift remained consistent at a 2% hydrogen concentration, indicating stable sensitivity of the sensor. This further demonstrates the suitability of the material for the detection of different hydrogen concentrations with good sensitivity performance. The data in Figure S4 (b), (c), and (d) show high repeatability and stability with good consistency of sensor performance over multiple uses with no performance degradation seen.

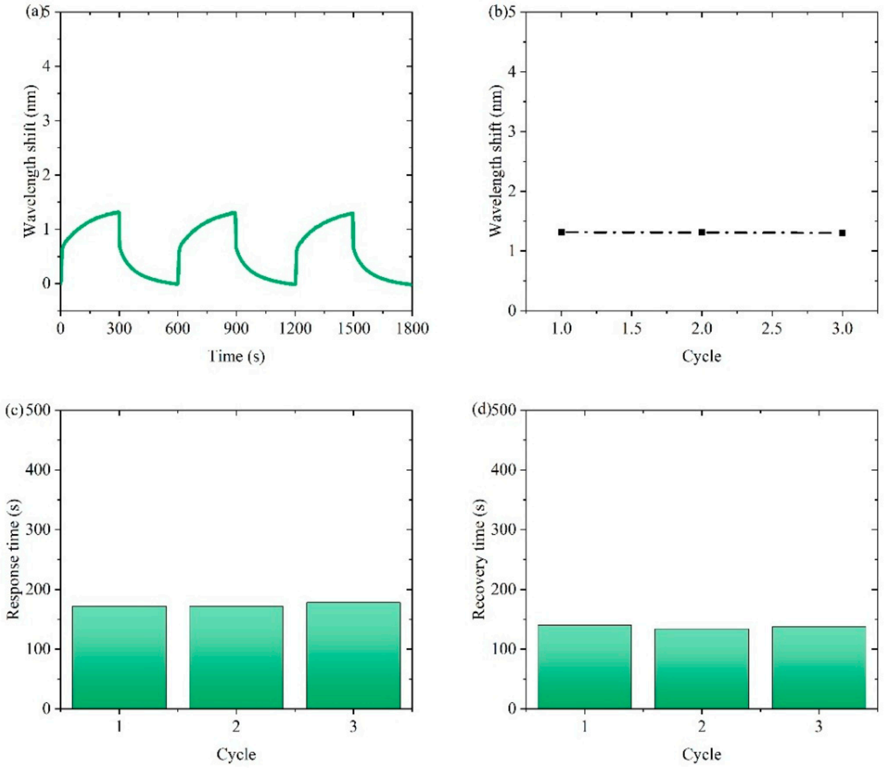

Figure S4. The results of the continuous repeatability of the sensor for 2% concentration of hydrogen. (a) The results of continuous testing. (b) The wavelength response for the three tests. (c) The response times for the three tests. (d) The recovery times for the three tests.

Figure S5 shows the results of the continuous repeatability of the sensor for a 2.5% hydrogen concentration. Figure S5 (a) shows the results of the continuous test, where the wavelength shift over time is shown in three cycles, with a consistent response for each test cycle and all of them recovering quickly to the baseline level after the peak. The curve pattern of the continuous test shows that the material still maintains a consistent response behavior under a high concentration of hydrogen, and there is no obvious degradation of the performance. Figure S5 (b) shows the wavelength response of the three tests, the maximum value of the wavelength shift is consistent in the three test cycles, and the average value is 2.165 nm. There is no obvious fluctuation or abnormality in the data, and the repeatability of the three tests is good. At a 2.5% hydrogen concentration, the sensor showed high repeatability in detecting hydrogen. The high repeatability is an indication of the stability of the sensor and is suitable for long-term use and multiple repeat test scenarios. Figure S5 (c) shows the response time for the three tests. The response time remained consistent throughout the three tests with an average of 183 seconds. The stability of the response time indicates that the response speed of the sensor is not affected by the increase in hydrogen concentration. Figure S5 (d) shows the recovery time for the three tests, the recovery time also remains consistent over the three tests with an average of 151 seconds. The stability of the recovery time indicates the reversibility of the material during hydrogen desorption and the integrity of the surface active sites. The fast recovery capability ensures that the sensor can be used at high frequencies without cumulative effects.

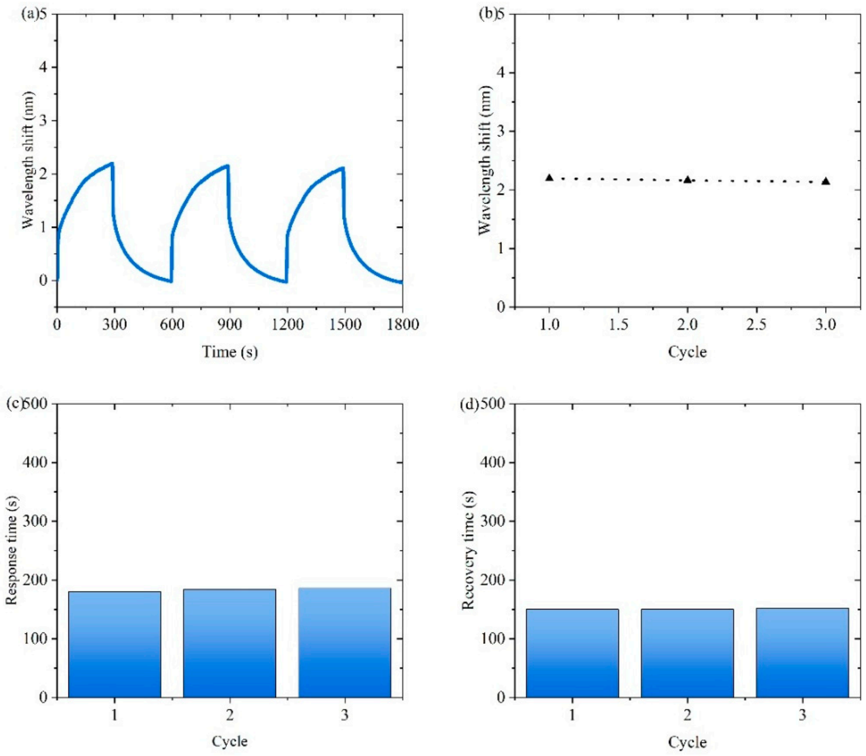

Figure S5. The results of the continuous repeatability of the sensor for 2.5% hydrogen concentration. (a) The results of the continuous test. (b) The wavelength response of the three tests. (c) The response time for the three tests. (d) The recovery time for the three tests.

Figure S6 shows the results of the continuous repeatability of the sensor for a 3% concentration of hydrogen. Figure S6 (a) shows the results of continuous testing, the

wavelength shift over time shows three cycles, the peak response and recovery curve of each cycle are of the same shape, and the peak is followed by a rapid recovery to the baseline level. The amplitude of the wavelength shift remains stable at a 3% hydrogen concentration, indicating that the sensor can still maintain good sensitivity at higher concentrations. The curve shape indicates that the material has good adsorption/desorption behavior, and no obvious drift or cumulative effect was observed. Figure S6 (b) shows the wavelength response of the three tests, the maximum value of the wavelength shift remains consistent, and the average value is 3.125 nm. There is no significant fluctuation or abnormality, and the data repeatability is very high. The high consistency of the wavelength shift indicates that the sensor has a stable response at the same concentration of hydrogen and the results are highly reproducible. Figure S6 (c) shows the response times for the three tests, which were generally consistent over the three tests with an average value of 190 seconds. Figure S6 (d) shows the recovery time for the three tests. The recovery time was consistent over the three tests with an average value of 146 seconds. The stability of the recovery time indicates that the material is well reversible during hydrogen desorption and the surface active sites are not depleted. The fast recovery capability supports high-frequency use of the sensor and is suitable for continuous detection environments.

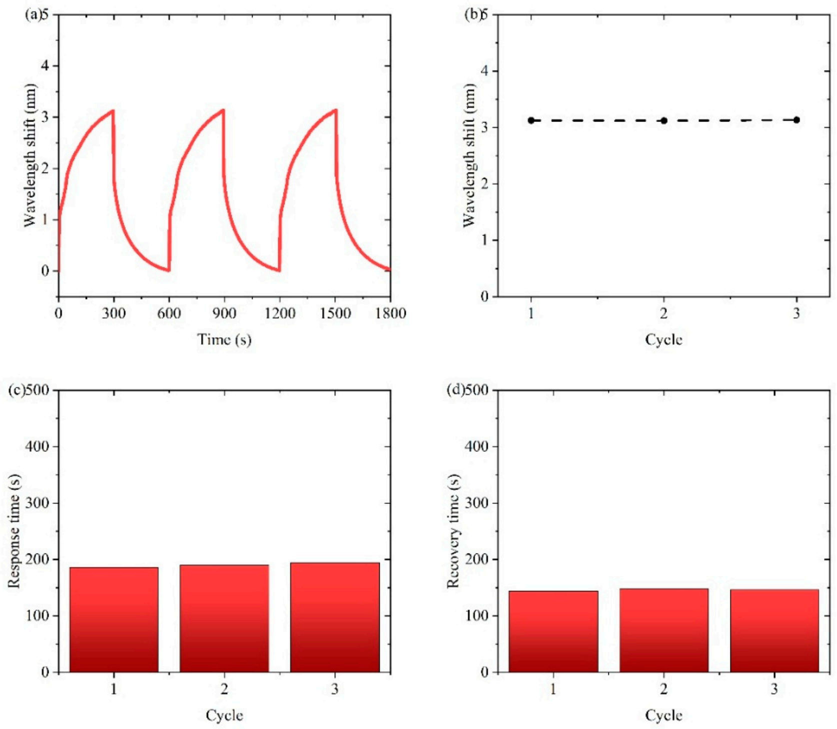

Figure S6. The results of the continuous repeatability of the sensor for 3% concentration of hydrogen. (a) The results of continuous testing. (b) The wavelength response of the three tests. (c) The response times for the three tests. (d) The recovery time for the three tests.

Figure S7 shows the results of the continuous repeatability of the sensor for a 3.5% concentration of hydrogen. Figure S7 (a) shows the results of the continuous test where the wavelength shift peaks in each cycle and then quickly returns to the baseline level. The shape of the response curve is consistent over the three test cycles, indicating stable sensor performance at a 3.5% hydrogen concentration. At a high (3.5%) hydrogen concentration, the sensitivity of the sensor remained constant and the magnitude of the

wavelength shift remained stable. The consistency of the curve morphology indicates that the dynamic adsorption/desorption behavior of the material was not affected by cumulative effects or high concentrations in successive tests. The sensor is suitable for continuous on-line testing in high-hydrogen-concentration environments. Figure S7 (b) shows the wavelength response of the three tests, and the wavelength shift maximum is averaged over the three tests at 4.015 nm. The fluctuation between the data is very small, which shows good repeatability. Figure S7 (c) shows the response time of the three tests, the response time is basically the same in the three tests, which is about 177 seconds. Figure S7 (d) shows the recovery time for the three tests. The recovery time is consistent across the three tests and is approximately 141 seconds. The stability of the recovery time indicates that the reversibility of the material during hydrogen desorption is good, and the surface active sites did not show any degradation of performance due to the effect of the high concentration of hydrogen.

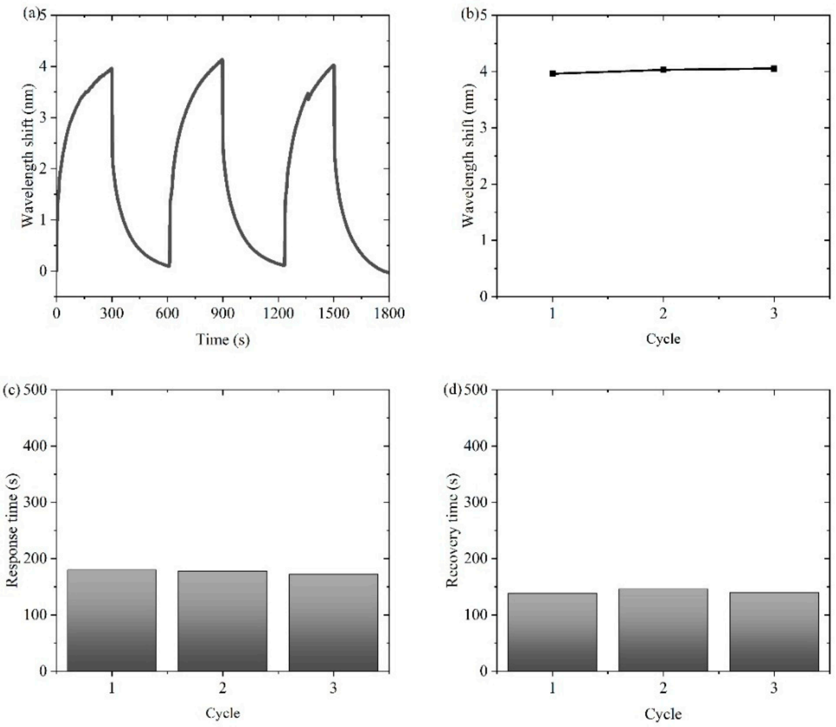

Figure S7. The results of the continuous repeatability of the sensor for a 3.5% concentration of hydrogen. (a) The results of the continuous test. (b) The wavelength response of the three tests. (c) The response time of the three tests. (d) The recovery time for the three tests.

Figure S8 shows the experimental data of the second channel of the fiber-optic hydrogen sensor with thermo-optic nanomaterials at a 0.5% concentration of hydrogen. Figure S8 (a) shows the results of continuous experiments. The wavelength shift versus time plot shows a stable response curve with multiple periodic fluctuations, a stable wavelength shift amplitude in each cycle, and a fast recovery of the wavelength after the peak, indicating that the sensor responds well to hydrogen concentration. The stable response of the sensor at a 0.5% hydrogen concentration indicates that it can accurately detect hydrogen changes at this concentration. The stabilization of the wavelength shift amplitude indicates that the adsorption and desorption process of hydrogen by the material is reversible and there is no long time drift or cumulative effect. Figure S8 (b) shows the wavelength response in three experiments. In the three experiments, the

wavelength shift remained consistent in each experiment with an average wavelength shift of 0.222 nm. Figure S8 (c) shows the response time in the three experiments. In the three experiments, the response time is basically consistent with an average response time of 52 seconds. There is no obvious response time fluctuation, indicating that the adsorption and detection of hydrogen by the sensor are fast and consistent. Figure S8 (d) shows the recovery time in the three experiments. The recovery time is also very consistent among the three experiments, with an average of 33 seconds, and the sensor is able to quickly recover from the hydrogen environment to the initial state.

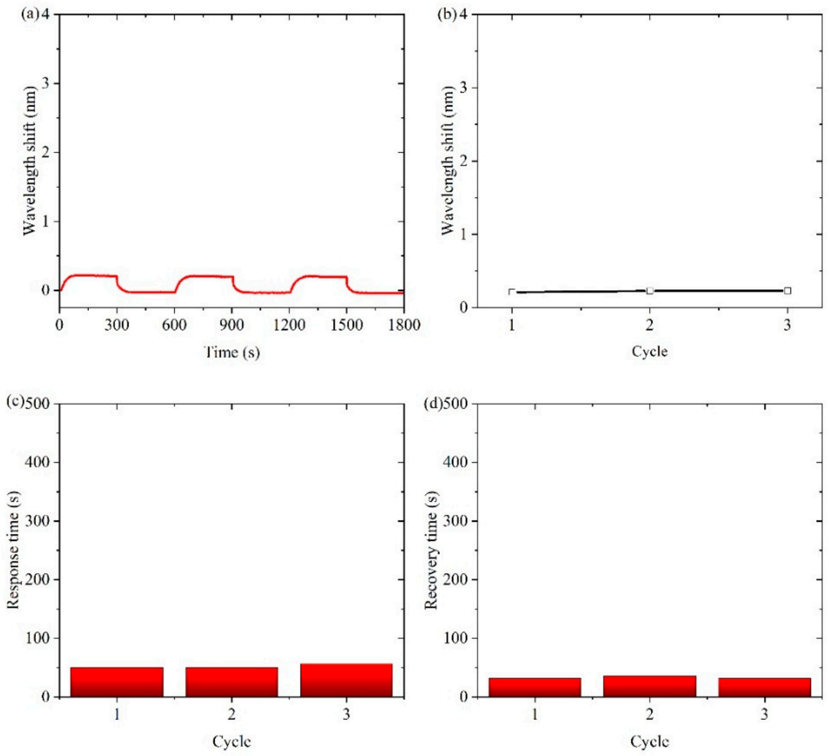

Figure S8. The experimental data of the second channel of the fiber-optic hydrogen sensor with thermo-optic nanomaterials at a 0.5% concentration of hydrogen. (a) The results of continuous experiments. (b) The wavelength response in three experiments. (c) The response time in the three experiments. (d) The recovery time in the three experiments.

Figure S9 shows the experimental data for the second detection channel of the hydrogen exothermic nanomaterial fiber-optic hydrogen sensor at a 1% concentration of hydrogen. Figure S9 (a) shows the results of continuous experiments. The figure shows the variation of the characteristic wavelength of the sensor with time. It can be seen in the figure that the wavelength change shows periodic fluctuations and the sensor is periodically exposed to hydrogen, indicating that the sensor is able to detect hydrogen stably. Figure S9 (b) shows the wavelength response in three experiments. The figure shows the wavelength variation in three experiments. The data in the graph show consistent wavelength changes in each experiment, which indicate that the sensor has good repeatability and reliability in detecting hydrogen in each cycle. The wavelength variation over the three experiments is very consistent, indicating good stability of the sensor over multiple cycles with an average wavelength response of 0.421 nm. Figure S9 (c) shows the response time over the three experiments. The figure shows that the response time remained roughly consistent over the three experiments, with an average

response time of 118 seconds. This indicates that the sensor responds faster to hydrogen and there is no significant increase in response time over multiple cycles and the response time of the sensor is stable. Figure S9 (d) shows the recovery time over the three experiments. Similar to the response time, the recovery time remained consistent over the three cycles with an average response time of approximately 65 seconds. This indicates that the sensor was able to recover to baseline quickly after hydrogen exposure, and the recovery time did not become longer with increasing cycles, suggesting that the sensor has good stability in terms of recovery capability.

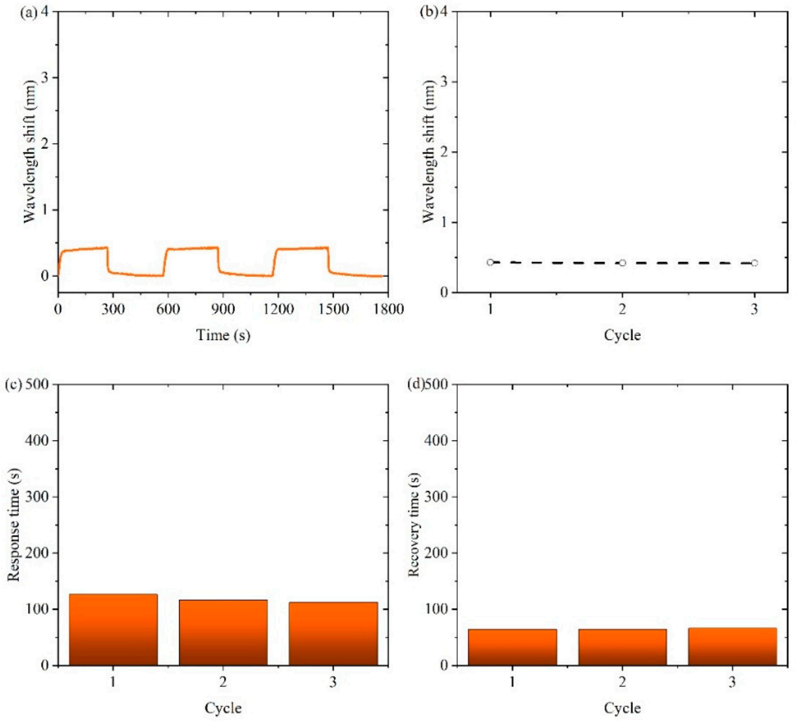

Figure S9. The experimental data for the second detection channel of the hydrogen exothermic nanomaterial fiber-optic hydrogen sensor at a 1% concentration of hydrogen. (a) The results of continuous experiments. (b) The wavelength response in three experiments. (c) The response time over the three experiments. (d) The recovery time over the three experiments.

In Figure S10, the experimental data of the second detection channel of the hydrogen exothermic nanomaterial fiber-optic hydrogen sensor at a 1.5% concentration of hydrogen are shown. Figure S10 (a) shows the results of the continuous experiment. The wavelength changes show periodic fluctuations, indicating that the sensor was exposed to hydrogen at each cycle. Figure S10 (b) shows the wavelength response in three experiments. The wavelength changes in each cycle are represented by triangle symbols in the graph, showing the repeatability of the wavelength response. The average wavelength response of the sensor to a 1.5% concentration of hydrogen was 0.642 nm. Figure S10 (c) shows the response time over the three experiments. The average response time for the three experiments was 118 seconds, indicating that the sensor responded relatively quickly to hydrogen and did not change significantly from cycle to cycle. Figure S10 (d) shows the recovery time in the three experiments. The recovery time is similar to the response time, with an average recovery time of approximately 65 seconds over the three experiments. This indicates that the sensor is able to quickly recover to the baseline state after exposure

to hydrogen, and the recovery time remains consistent over multiple cycles, showing the stability of the sensor in terms of recovery capability.

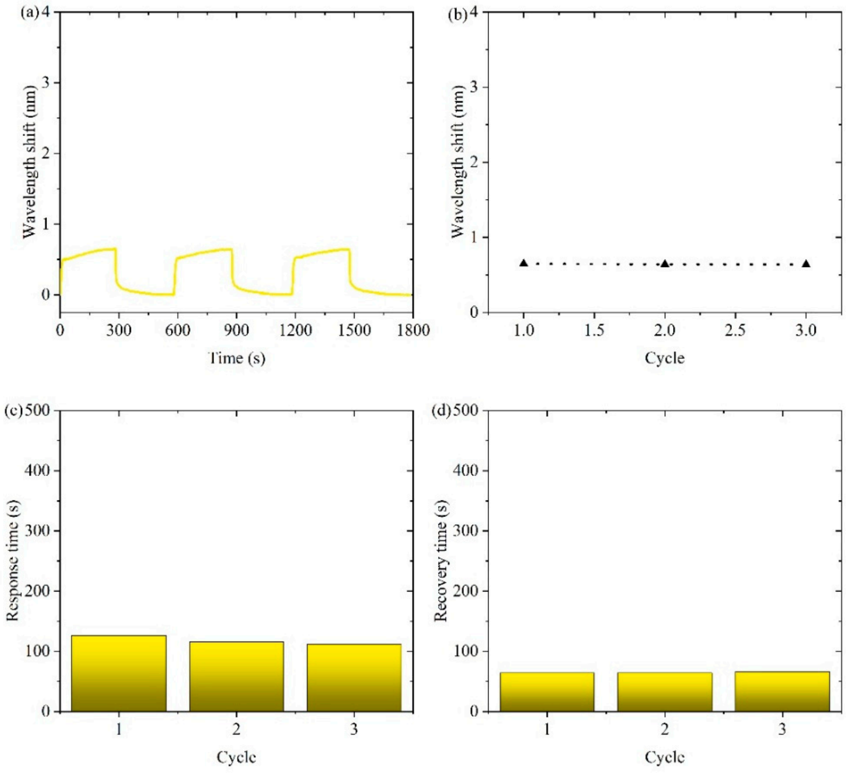

Figure S10. The experimental data of the second detection channel of the hydrogen exothermic nanomaterial fiber-optic hydrogen sensor at a 1.5% concentration of hydrogen. (a) The results of the continuous experiment. (b) The wavelength response in three experiments. (c) The response time over the three experiments. (d) The recovery time in the three experiments.

Figure S11 shows the experimental data for the second detection channel of the hydrogen exothermic nanomaterial fiber-optic hydrogen sensor at a 2% concentration of hydrogen. Figure S11 (a) shows the results of the continuous experiment. The sensor has a reversible and stable response to a 2% concentration of hydrogen. Figure S11 (b) shows the wavelength response in three experiments. The wavelength response in each cycle is indicated by the inverted triangle symbols, and the wavelength response is more consistent, showing good repeatability. The wavelength response remained stable between cycles, indicating that the sensor was able to detect hydrogen stably each time it was exposed to hydrogen, and there was no significant difference in performance across the three cycles. The average wavelength response was 0.845 nm. Figure S11 (c) shows the response time over the three experiments. The average response time is 135 s. The sensor has a stable response rate and good responsiveness to detect 2% hydrogen. There is no significant increase in response time compared to the low-concentration experiment, showing the adaptability of the sensor to different concentrations of hydrogen. Figure S11 (d) shows the recovery time in three experiments. The average recovery time was about 83 seconds, which did not change significantly over the three cycles. This indicates that the sensor is able to quickly recover to the baseline state after exposure to hydrogen, and the recovery time is highly consistent between different cycles, demonstrating its excellent recovery ability and stability.

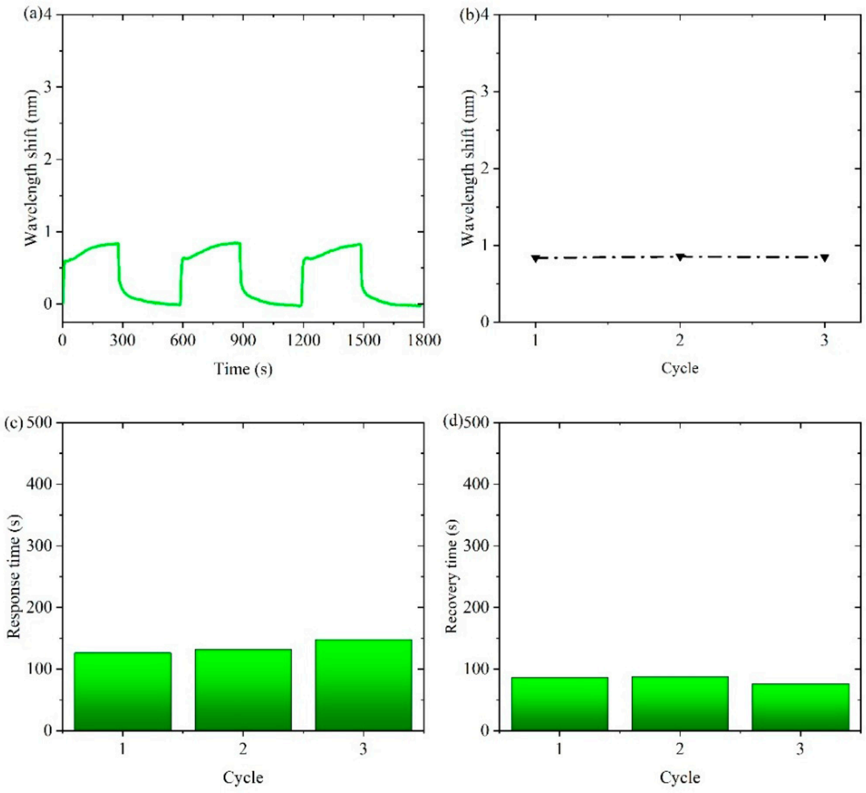

Figure S11. The experimental data for the second detection channel of the hydrogen exothermic nanomaterial fiber-optic hydrogen sensor at a 2% concentration of hydrogen. (a) The results of the continuous experiment. (b) The wavelength response in three experiments. (c) The response time over the three experiments. (d) The recovery time in three experiments.

Figure S12 shows the experimental data for the second detection channel of the hydrogen exothermic nanomaterial fiber-optic hydrogen sensor at a 2.5% concentration of hydrogen. Figure S12 (a) shows the results of continuous experiments. The wavelength change shows periodic fluctuations, indicating that the sensor is able to respond stably to hydrogen during each cycle. The wavelength variation remains within a relatively stable range over time. This indicates that the sensor is able to respond well to hydrogen at a concentration of 2.5% and the response is stable. Figure S12 (b) shows the wavelength response in three experiments. The wavelength change in each cycle is indicated by the diamond symbols, and the wavelength response is very consistent over the three experiments and the change remains within a relatively stable range. This indicates that the sensor was able to respond stably to hydrogen over multiple experimental cycles without large fluctuations, showing good repeatability, with an average wavelength response of 1.095 nm. Figure S12 (c) shows the response time over the three experiments. The average response time is about 171 seconds, and the response time variation in the three experiments is very small. It shows that the response speed of the sensor at a 2.5% concentration of hydrogen is stable, and the response time is not significantly prolonged with the increase in the experimental period, which indicates that the sensor is able to respond rapidly to hydrogen in different periods. Figure S12 (d) shows the recovery time in three experiments. The average recovery time is about 61 seconds and does not change significantly over the three cycles. This indicates that the sensor is able to quickly recover to the baseline state after hydrogen exposure, and the recovery time is stable without significant changes, indicating good recovery ability.

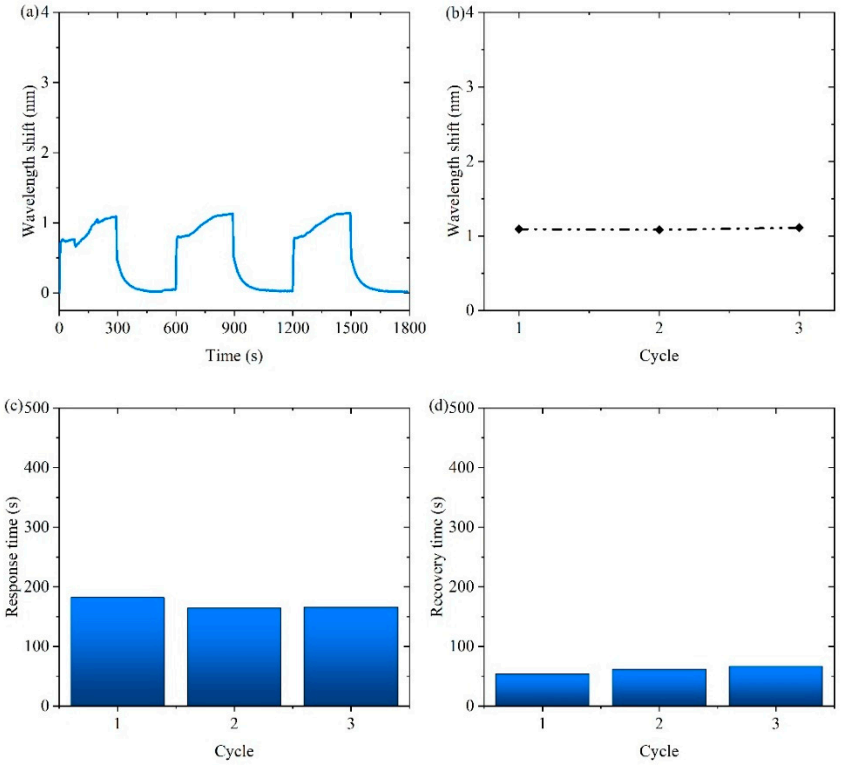

Figure S12. The experimental data for the second detection channel of the hydrogen exothermic nanomaterial fiber-optic hydrogen sensor at a 2.5% concentration of hydrogen. (a) The results of continuous experiments. (b) The wavelength response in three experiments. (c) The response time over the three experiments. (d) The recovery time in three experiments.

Figure S13 shows the experimental data for the second detection channel of the hydrogen exothermic nanomaterial fiber-optic hydrogen sensor at a 3% concentration of hydrogen. Figure S13 (a) shows the results of continuous experiments. The wavelength changes show periodic fluctuations, showing that the sensor responds significantly in each cycle. Figure S13 (b) shows the wavelength response in three experiments. The wavelength changes in each cycle are indicated by triangular symbols, and the wavelength responses in the figure are consistent and stable. It shows that the wavelength response of the sensor is reproducible and stable over multiple experimental cycles, and it can reliably detect hydrogen without large fluctuations, with an average wavelength response of 1.207 nm. Figure S13 (c) shows the response time over the three experiments. The average response time is about 169 s, and the response time varies very little in the three experiments. This shows that the response speed of the sensor at a 3% concentration of hydrogen is very stable and there is no obvious change between different cycles, indicating that its response ability is more balanced and adaptable. Figure S13 (d) shows the recovery time in the three experiments. The average recovery time was about 93 seconds and did not change significantly over the three cycles. This indicates that the sensor is able to quickly recover to the baseline state after hydrogen exposure, and the recovery time is stable without significant changes, showing a good recovery capability.

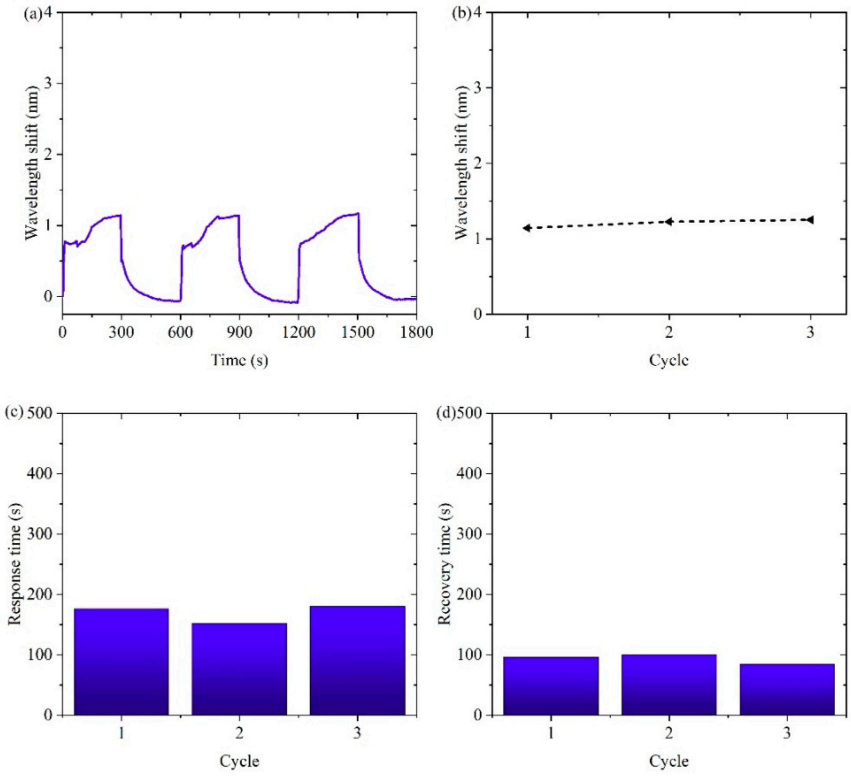

Figure S13. The experimental data for the second detection channel of the hydrogen exothermic nanomaterial fiber-optic hydrogen sensor in 3% concentration of hydrogen. (a) The results of continuous experiments. (b) The wavelength response in three experiments. (c) The response time over the three experiments. (d) The recovery time in the three experiments.

Figure S14 shows the experimental data for the second detection channel of the hydrogen exothermic nanomaterial fiber-optic hydrogen sensor at a 3.5% concentration of hydrogen. Figure S14 (a) shows the results of continuous experiments. The wavelength change shows periodic fluctuations, showing a stable response of the sensor in each cycle. The highest value of the amplitude of the wavelength change increased from the previous experimental cycles. This indicates that the sensor response at a 3.5% hydrogen concentration has increased in amplitude and is stable. As the hydrogen concentration increases, the sensor shows a strong response. Figure S14 (b) shows the wavelength response over the three experiments. The wavelength changes are indicated by the triangular symbols, and the wavelength responses of the three experiments are consistent and well reproducible, and the magnitude of the wavelength changes is basically the same. This indicates that the sensor is able to detect hydrogen reliably in different cycles and the wavelength response is highly stable, with an average wavelength response of about 1.626 nm. Figure S14 (c) shows the response time in the three experiments. The average response time is about 177 s, and the response time varies less in the three experiments. The good stability of the response time indicates that the sensor responded quickly to the 3.5% hydrogen concentration and was able to maintain a consistent response time. Figure S14 (d) shows the recovery time in the three experiments. The average recovery time was approximately 138 seconds but increased slightly as the cycle progressed. This indicates that the sensor was able to recover to the baseline state relatively quickly after hydrogen exposure, but the recovery time may have increased slightly as the hydrogen concentration increased.

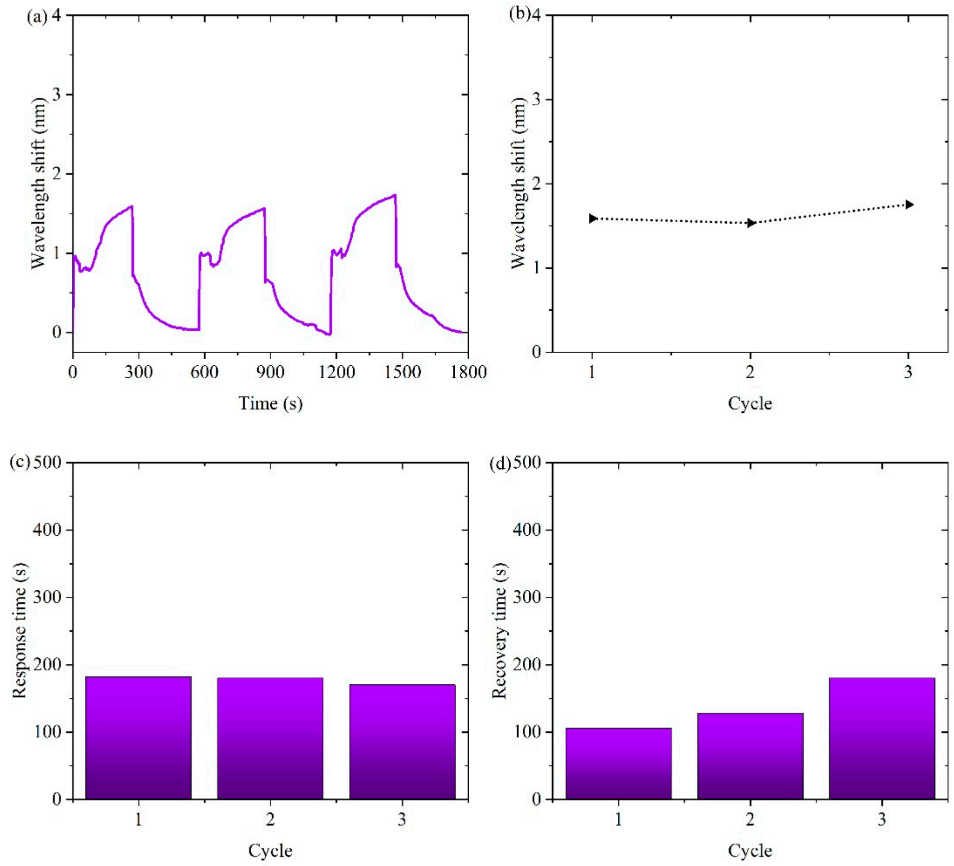

Figure S14. The experimental data for the second detection channel of the hydrogen exothermic nanomaterial fiber-optic hydrogen sensor at a 3.5% concentration of hydrogen. (a) The results of continuous experiments. (b) The wavelength response over the three experiments. (c) The response time in the three experiments. (d) The recovery time in the three experiments.

We propose a fiber-optic hydrogen sensing system based on thermo-optic nanomaterials as shown in Figure S15. The sensing system contains a Broadband Source, and the output light is split into two independent hydrogen sensing channels through a Fiber Coupler. The transmitted light, after passing through the two sensors, enters the Fiber Optical Switch, through which we select the optical signal that enters the Optical Spectrum Analyzer. Thus, the sensing system uses only one light source and one spectrometer. We control the spectrometer through a computer to collect, analyze, and save optical signals.

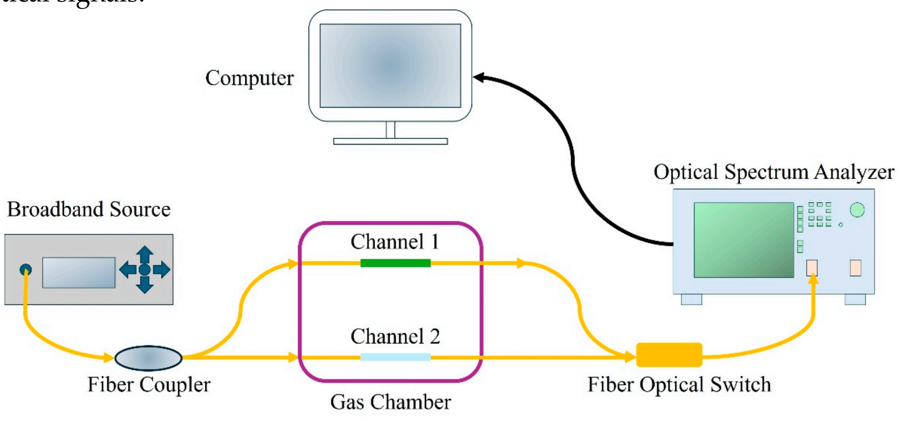

Figure S15. Dual-channel fiber-optic hydrogen sensing test system based on thermo-optic nanomaterials.

We further processed the data in Figure 4 (b) to compare the response in the phase of gradual increase and gradual decrease of hydrogen concentration. As can be seen from Figure S16, the characteristic wavelength shifts of the sensor during the increasing (Increase, indicated by black squares) and decreasing (Decrease, indicated by red dots) phases of hydrogen concentration are almost coincident, and the gap between the two curves is extremely small. This indicates that the sensor response is basically the same for hydrogen adsorption and desorption at the same concentration point. Since the curves of the rising and falling processes basically overlap, the response of the sensor during the change in hydrogen concentration is highly reversible, indicating that there is almost no hysteresis effect in the hydrogen absorption and desorption processes. This phenomenon implies that nanomaterials have excellent kinetic properties in the hydrogen adsorption/desorption process, and the fiber-optic hydrogen sensor exhibits good response reversibility.

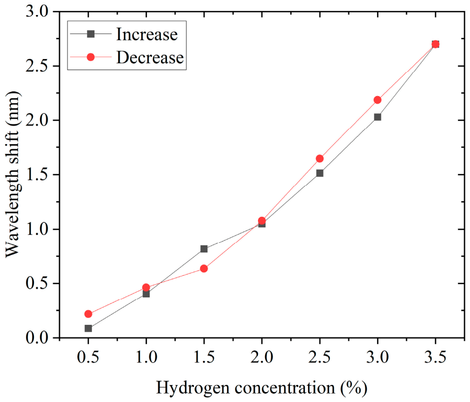

Figure S16. The characteristic wavelength shifts correspond to the hydrogen concentration.

We further processed the data in Figure 8 (b) for the hydrogen-sensitive response of channel 2. A comparison of the responses for the gradually increasing and gradually decreasing phases of hydrogen concentration was obtained as shown in Figure S17. The characteristic wavelength shifts of channel 2 of the sensor during the rising (Increase, indicated by the blue squares) and falling (Decrease, indicated by the green dots) phases of hydrogen concentration almost overlap, and the gap between the two curves is extremely small. This indicates that the sensor response is essentially the same for hydrogen absorption and desorption at the same concentration point. The curves for the rising and falling phases largely overlap, indicating that the response of the sensor is highly reversible. Channel 2 of this fiber-optic hydrogen sensor has almost no hysteresis and shows good response reversibility.

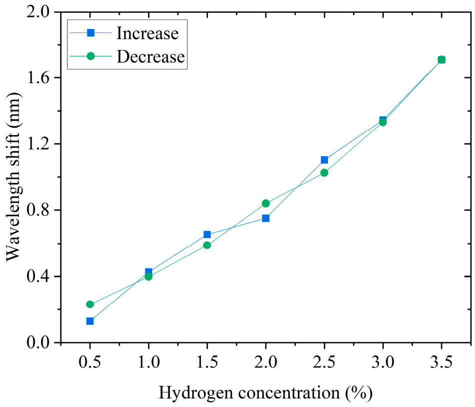

Figure S17. The effect of hydrogen concentration changes on the wavelength shifts of the sensor.

To investigate the temperature-sensitive characteristics of the sensor, we tested the response of the two channels of the sensor to changes in ambient temperature separately, as shown in Figure S18. Figure S18 (a) shows the response of channel one to ambient temperature changes, and the spectral response of the sensor was tested in the range of 40 °C to 80 °C. The characteristic spectrum redshifts as the temperature gradually increases. Figure S18 (b) shows the response of channel two to ambient temperature changes, where the spectral response of the sensor was tested in the range of 38 °C to 79 °C. Like channel one, the characteristic wavelength gradually becomes larger as the temperature gradually increases. The temperature test data further corroborate the working principle of the sensor, where the nanomaterials undergo hydrogen exotherm in hydrogen gas, causing the sensor spectrum to redshift.

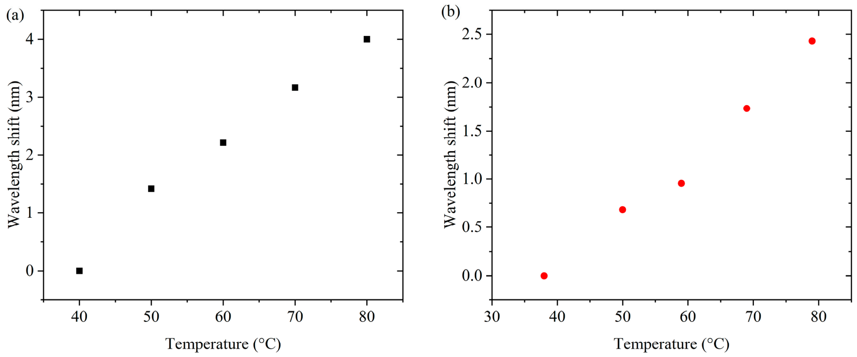

Figure S18. Response of the two channels of the sensor to changes in ambient temperature. (a) The response of channel one to ambient temperature changes. (b) The response of channel two to ambient temperature changes.

Disclaimer/Publisher's Note: The statements, opinions and data contained in all publications are solely those of the individual author(s) and contributor(s) and not of MDPI and/or the editor(s). MDPI and/or the editor(s) disclaim responsibility for any injury to people or property resulting from any ideas, methods, instructions or products referred to in the content.
